# Impact of the *FTO* rs9939609 risk allele on subcutaneous adipose tissue fatty acid composition in adults with obesity class 2 and 3

**DOI:** 10.1371/journal.pone.0351698

**Published:** 2026-06-17

**Authors:** Ingrid Løvold Mostad, Valdemar Grill, Sissel Skarra, Ann Kristin Hjelle de Soysa, Barbara Fielding

**Affiliations:** 1 Department of Nutrition and Speech-Language Therapy, Clinic of Rehabilitation, St. Olavs hospital - Trondheim University Hospital, Trondheim, Norway; 2 Department of Clinical and Molecular Medicine, Faculty of Medicine and Health Sciences, Norwegian University of Science and Technology, Trondheim, Norway; 3 Department of Circulation and Medical Imaging, Faculty of Medicine and Health Sciences, Norwegian University of Science and Technology, Trondheim, Norway; 4 Outpatient Obesity Clinic, Clinic of Surgery, St. Olavs hospital - Trondheim University Hospital, Trondheim, Norway; 5 Faculty of Health and Medical Sciences, University of Surrey, Guildford, Surrey, United Kingdom; University of Diyala College of Medicine, IRAQ

## Abstract

The *FTO* rs9939609 risk allele is linked to risk of obesity. Whether causality involves fatty acid (FA) metabolism remains to be fully investigated in adults with obesity. We tested for associations of the risk allele with the FA composition of android and gynoid subcutaneous adipose tissue. We recruited 95 participants with obesity class 2 and 3 and without diabetes, median BMI 42.8 (25^th^, 75^th^ percentiles: 39.5, 46.5) kg/m^2^. Participants carried no (TT, n = 33), one (AT, n = 31), or two (AA, n = 31) copies of the *FTO* risk allele. Biopsies were obtained by aspiration and total FA composition determined by gas chromatography-mass spectrometry (GC-MS). In the cohort overall, there were no significant genotype associations with any single FA. In males with the TT allele, mass of oleic acid (18:1n-9) in the gynoid depot was higher compared with the AT allele when corrected for depot size. We interpret these findings with caution due to the small numbers of males with the TT genotype. Disregarding genotype, in the cohort overall, proportions of saturated FAs were higher, and proportions of monounsaturated FAs lower in android versus gynoid adipose tissue, confirming previous studies. We found previously unreported sex-related differences in FA composition (weight %) and content (weight % corrected for depot mass). Our findings on *FTO* genotype are generally negative; observations to the contrary require confirmation as does the non-genetic and novel results on sex-related differences.

## Introduction

Variants in the *FTO* locus promote obesity through mechanisms related to appetite and satiety [1–4]. *FTO* is expressed in many cell types, and acts as a powerful demethylase towards multiple methylated RNA substrates [5]. Furthermore, the demethylase activity has been shown to be regulated by NADP, and deletion of *FTO* has been found to block NADP-enhanced obesogenesis in 3T3-L1 preadipocytes [6]. This suggests that mechanisms not directly related to satiety participate in the impact of *FTO* on obesogenesis.

Regarding effects of *FTO* that are not directly related to appetite, it seems pertinent to focus on potential effects on lipid metabolism in adipose tissue. The fatty acid (FA) composition of the diet is to some extent reflected in the composition of FA in adipose tissue [7] and differs according to depots in men and women [8,9]. Adipose tissue FA composition has been reported to be different in obesity compared with controls [10], and is modified by metabolic pathways, such as lipolysis [11] and pathways of fatty acid synthesis and desaturation [8]. The FA composition of adipose tissue in turn, has effects on signalling pathways via gene transcription and membrane fluidity [12], which, as well as obesity, contribute to metabolic dysfunction, such as chronic inflammation [13].

Genetic effects on metabolism that were not related to BMI and affected by the *FTO* obesogenic rs9939609 risk allele have been reported by Ponce-Gonzales [14] and by ourselves [15–19]. Any coupling of *FTO* risk alleles to the composition of FA in adipose tissue has so far not been tested. Several studies report that larger whole body adiposity is associated with the AA genotype of the *FTO* rs9939609 [20–22]. However, the effect of a risk genotype on metabolic parameters must be separated from a generalized obesity-promoting effect of *FTO*. We fail to find that such separation was reported in the previous studies [20–22]. Whether an obesity risk allele exerts preferential effects on the size (rather than the FA composition) of specific adipose depots is therefore unsettled. Hence, investigations into a possible impact of an *FTO* obesity risk allele on FA composition taking into account the size of specific depots are relevant.

We hypothesised that adipose tissue FA composition (weight %) and content (g per depot) would be affected by *FTO* polymorphisms, and aimed to measure the FA composition and depot size of android and gynoid subcutaneous adipose tissue of females and males with obesity, with *FTO* rs9939609 variant alleles AA, AT and TT.

## Materials and methods

### Participants and study design

**Fig 1:**
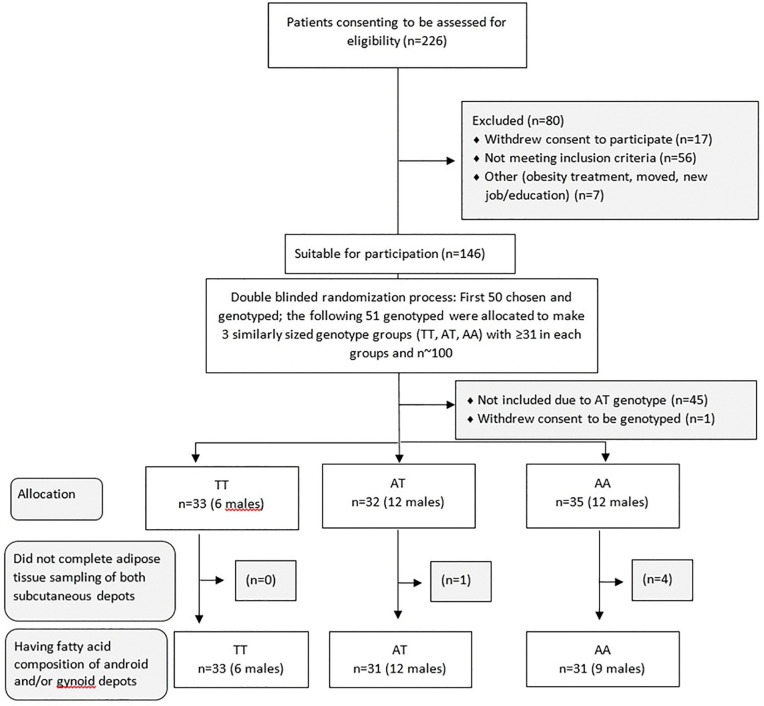
Flow diagram, participant selection, and group allocation. Adapted from de Soysa *et al* [15].

The study population has been described [15]. Briefly, in this cross-sectional metabolic and genetic observation study we included adults 20 y or older with BMI ≥ 35 kg/m^2^ without a diagnosis of diabetes. Individuals recruited over a two-year period from 2013 to 2015 had newly been referred to a university hospital outpatient obesity clinic for adults with obesity class 2 or 3. We aimed for 100 participants in the study with an equal number of participants that carried no (TT), one (AT), or two (AA) copies of the *FTO* rs9939609 risk allele. The DNA extraction and the genotyping of *FTO* rs9939609 have been described [15]. Enrolment and genotype allocations are shown in Fig 1. The selection of participants was blinded to investigators and participants. The study was conducted according to the guidelines laid down in the Declaration of Helsinki and all procedures involving research study participants were approved by the Regional committee for medical and health research in Central Norway, Regional komité for medisinsk og helsefaglig forskningsetikk, Midt-Norge, REK midt, registration number 2013/642. The director of the clinic approved the study, on behalf of the research responsible institut‌‌ion. All volunteers provided written, signed informed consent to participate.

### Measurements

#### Anthropometry and body composition.

Height and weight were recorded (in underwear) to the nearest 0.1 cm and to the nearest 0.1 kg respectively (Seca 285 wireless measuring station, Hamburg Germany), after a 10 h overnight fast on the day of adipose tissue sampling. Body mass index (BMI) was calculated by kg weight/m height^2^. Body composition was measured the same day by dual energy x-ray absorptiometry (DXA) (Holigic, Inc., Apex Software, Bedford, MA, USA). We measured head, trunk and legs for the DXA scan of the body; we could not fully scan the arms because the equipment was not designed to accommodate the obesity range in our study. Fat mass was defined as kg fat mass of trunk, left and right lower extremities. Visceral and gynoid fat mass were estimated in g by DXA. Subcutaneous android fat mass was calculated by subtracting the visceral fat mass from the total android fat mass. We analyzed the FA content in two ways: individual FA as weight %, and individual FA as total g per mass of each fat depot.

#### Sampling adipose tissue.

Research nurses performed the fat biopsies on the participants, using a percutaneous needle [23]. Aseptic techniques were used throughout. In brief, subcutaneous adipose tissue samples were aspirated from the upper outer quadrant of the buttock (gynoid depot) and 4–7 cm lateral to the umbilicus (android depot). The aspirate was poured onto a sterile nylon filtration tissue (sifting fabric, mesh opening 250 µm, Sefar Nitex 03–250/50, Filter Media & Filter Komponenter, Solna, Sweden) over a beaker. The aspirates were washed with sterile 0.9% sodium chloride. Any blood clots were removed with forceps before the aspirates were snap-frozen in liquid nitrogen for 60 sec.

We obtained paired biopsies of at least 150 mg each from 91 participants. Due to technical difficulties, we obtained biopsies from only one depot (android or gynoid) from four participants. Biological samples in our study were registered in the official biobank in Central Norway, Biobank1®.

#### Extracting fat from adipose tissue samples.

We obtained the lipid fraction of the adipose tissue using an adaptation of a method used for RNA extraction described in Hodson L *et al* [23]. Briefly, we isolated 50–150 mg of tissue, lysed in 700 µl lysis buffer and extracted with 700 µl phenol/chloroform. The phase separation was performed at 4 °C for 20 minutes. The lipid fraction was frozen and stored at −80 °C until analysis of the FA.

#### Gas chromatography-mass spectrometry (GC-MS).

From each thawed lipid sample 50 µl was pipetted into labelled glass 12 ml round bottom screw cap tubes and processed as previously reported, as supplementary data in Hodson L *et al* [23], using cod liver oil (Möllers tran, Oslo, Norway, processed 07.06.2016) as a quality control. Briefly, total fat was extracted into 2:1 chloroform methanol and evaporated to dryness under nitrogen. After reconstitution in toluene, FA methyl esters were prepared using acidic methanol and the solvent layer evaporated to dryness and reconstituted into 8 ml chloroform, and 200 µl volumes were transferred to GC vials for analysis. The FAME control standards used were Std B FAME Mix C4-C24 (Sigma-Aldrich, 100 mg, product 18919−1AMP) and FAME Mix RM-6 (Sigma-Aldrich, 100 mg, product 07631−1AMP). Samples were run on an Agilent GC-MS which was composed of a GC system 7890A, with MS detector 5975C, and autoinjector 7683B (Agilent Technologies, Inc., Santa Clara (USA)). The GC was equipped with a DB-23 J&W GC Column 60 m x 0.250 mm x 0.15 µm (Agilent technologies), with an injection volume of 0.2 µl. The oven’s starting temperature was 100 °C for 3 min, a temperature ramp of 25 °C per min to 200 °C for 12.5 min followed by 25 °C per min to 230 °C, a temperature which was upheld for 10 min. The samples were run on scan mode and identified by reference to the retention time of standards as well as by reference to the mass spectra. The proportion of each specific FA was determined from the peak areas of the total ion chromatograms and expressed as weight %. We also used the proportion of each FA and the mass of each subcutaneous depot in g, to calculate the total mass of each FA in g. The non-detectable peaks were set to zero. Because we lacked the DXA scan of fat mass variables from one participant, the calculation of each FA as g per depot are given for one participant less, than for the FA expressed as weight %. The correct number of participants is clearly marked in the header rows for each relevant table.

### Statistics

We performed statistics in IBM SPSS Statistics for Windows, Version 30.0. Armonk, NY: IBM Corp, released 2025. Summary statistics are presented with medians and 25^th^ and 75^th^ percentile values due to the presence of outliers and skewed data. We used the Independent-samples Kruskal-Wallis’s method including the Bonferroni correction for testing the null hypothesis that the distribution of the variables was the same across the three different genotypes, and the Independent-samples Mann-Whitney for testing that the distribution of the variables was the same across the two categories of sex. For testing depot differences (android versus gynoid) for median values of individual FAs and the groups of SFA (saturated FA), MUFA (monounsaturated FA) and PUFA (polyunsaturated FA), we used the Related-samples Wilcoxon signed rank test. A p*-*value < 0.05 was considered significant. When comparing effects of genotype with pairwise comparisons, we reported Bonferroni corrected p*-*values.

## Results

### Study population

Subcutaneous android and gynoid adipose tissue samples were obtained from 95 participants, all but one were Caucasian, n = 91 with paired samples. Blood glucose was within limits of normality [15,19]. Median (25^th^, 75^th^ percentiles) BMI was 42.8 (39.5, 46.5) kg/m^2^. Frequencies for genotypes were, for homozygote non-risk (TT), n = 33, for heterozygotes (AT), n = 31 and for homozygote risk alleles (AA), n = 31.

The distributions of age, weight, BMI, total -, visceral -, android – and gynoid fat mass were not different across categories of genotype for the whole cohort (**Table 1**). Female participants (n = 68) had lower median weight, visceral and android fat mass, and a higher gynoid fat mass compared with males (n = 27) (S1 Table). Age, weight, BMI, total -, visceral -, android – and gynoid fat mass did not differ across genotypes in females. Age was lower in males displaying the non-risk TT genotype compared with the AT and AA genotypes (Table 1, footnote *c*). Males with the TT genotype had a lower visceral fat mass than the AA genotype and a higher gynoid fat mass than the AT genotype, whereas no such differences were detected between the AT and AA genotypes (Table 1). The group of males with the non-risk TT genotype is very small (n = 6), which means that their results must be interpreted with caution.

**Table 1:** Characteristics of participants by genotype.

	All n = 95		TT n = 33		AT n = 31		AA n = 31		
			Females n = 27^*a*^		Females n = 19		Females n = 22		
			Males n = 6		Males n = 12		Males n = 9		
	Median	25^th^, 75^th^ percentiles	Median	25^th^, 75^th^ percentiles	Median	25^th^, 75^th^ percentiles	Median	25^th^, 75^th^ percentiles	P^*b*^
Age, year, n = 95	42	31, 50	39	30, 47	43	36, 51	44	31, 54	*.272* ^*c*^
Weight, kg, n = 95	120.9	109.5, 140.0	119.2	106.0, 141.3	127.1	109.5, 142.3	123.9	110.1, 136.8	*.897*
Females, n = 68			116.2	104.5, 134.6	117.9	103.7, 135.9	119.2	107.9, 128.2	*.965*
Males, n = 27			155.6	144.4, 165.3	140.7	122.5, 152.3	147.1	128.0, 155.3	*.196*
Body mass index, kg/m^2^, n = 95	42.8	39.5, 46.5	41.7	38.2, 46.6	41.1	37.8, 45.4	43.2	40.5, 47.4	*.367*
Females, n = 68			40.9	37.3, 46.5	40.7	37.8, 45.4	42.9	40.3, 46.5	*.478*
Males, n = 27			46.0	43.3, 47.3	42.6	37.8, 46.2	43.4	40.4, 49.4	*.392*
Visceral fat mass, g, n = 94	756	556, 947	633	538, 856	772	607, 937	852	630, 1062	*.103*
Females, n = 67			714	526, 873	716	567, 828	722	550, 954	*.821*
Males, n = 27			564	517, 660	960	802, 1065	1067	888, 1630	*.013* ^*d*^
Android fat mass, g, n = 94	4259	3228, 4897	4346	3440, 5302	3718	3015, 4985	4282	3446, 4773	*.836*
Females, n = 67			3935	2981, 4692	3343	3015, 4643	4259	3272, 4487	*.895*
Males, n = 27			5922	4937, 6164	4734	2913, 5280	4310	3685, 5520	*.113*
Gynoid fat mass, g, n = 94	7910	6410, 9528	8575	7585, 9693	7340	6030, 9480	7420	6010, 9440	*.239*
Females, n = 67			8575	7440, 9763	8480	6760, 10680	8040	6888, 10308	*.941*
Males, n = 27			8835	7333, 9728	5735	4618, 7663	5600	5030, 7180	*.036* ^*e*^
Fat mass, kg, n = 94	46.1	38.8, 53.7	47.4	40.2, 55.4	44.4	37.3, 53.5	47.0	39.9, 53.6	*.497*
Females, n = 67			45.6	39.7, 52.5	44.7	37.3, 58.0	46.4	40.4, 54.0	*.741*
Males n = 27			55.0	48.4, 59.6	43.2	33.9, 52.2	47.0	38.9, 54.0	*.099*

^*a*^For the four fat mass variables, n=26 for females.

^*b*^P represents uncorrected genotype difference.

^*c*^Age: females p-value 0.900; males p-value 0.007 with Bonferroni corrected p-values of pairwise genotype comparisons 0.041 for TT vs AT, 1.000 for AT vs AA and 0.006 for TT vs AA.

^*d*^Visceral fat, males: Bonferroni corrected p-values of pairwise genotype comparisons were 0.131 for TT vs AT, 0.647 for AT vs AA and 0.010 for TT vs AA.

^*e*^Gynoid fat mass, males: Bonferroni corrected p-values of pairwise genotype comparisons were 0.043 for TT vs AT, 1.000 for AT vs AA and 0.090 for TT vs AA.

### Effect of genotype on FA composition (weight %)

In the cohort overall, we found no genotype effects for individual FAs in either adipose tissue depot (**Table 2**). We note that total MUFA in the android depot was significantly affected overall by genotype (Table 2) and had a borderline higher proportion in the TT compared with AT genotype after Bonferroni correction (p = 0.057, Table 2, footnote *c*). We found no significant effects of genotype when testing for a difference between the android and gynoid depots for SFAs, MUFAs and PUFAs (Table 2). Exploring each sex separately, we found no genotype differences among females in either depot, but in males there was a higher proportion of the minor elaidic acid (18:1n-9t) in the non-risk TT in the gynoid depot, and a lower proportion of the arachidonic acid (20:4n-6) in the android depot, compared with the AT genotype (**Table 3**, footnotes *b*, *c*).

**Table 2:** Effect of genotype on fatty acid composition (weight %) of android and gynoid subcutaneous adipose tissue.

Fatty acids in adipose tissue subcutaneous depots	TT n = 31 (android), n = 33 (gynoid		AT n = 31 (android), n=30 (gynoid)		AA n = 31 (android), n=30 (gynoid)		Within each depot	Between depots
	Females n = 25 (android), n=27 (gynoid)		Females n = 19 (android, gynoid)		Females n = 22 (android), n=21 (gynoid)			
	Males n = 6 (android, gynoid)		Males n = 12 (android), n=11 (gynoid)		Males, n = 9 (android, gynoid)			
	Median weight %	25^th^, 75^th^ percentiles	Median weight %	25^th^, 75^th^ percentiles	Median weight %	25^th^, 75^th^ percentiles	P^*a*^	P^*b*^
Lauric acid, 12:0 (android)	0.24	0.00, 0.43	0.00	0.00, 0.36	0.34	0.00, 0.47	*.400*	*.426*
Lauric acid, 12:0 (gynoid)	0.00	0.00, 0.40	0.00	0.00, 0.30	0.00	0.00, 0.38	*.632*	
Myristic acid, 14:0 (android)	2.71	2.28, 2.94	2.68	2.43, 3.00	2.64	2.33, 2.79	*.638*	*.729*
Myristic acid, 14:0 (gynoid)	2.23	2.02, 2.56	2.30	2.02, 2.71	2.28	1.87, 2.55	*.774*	
Pentadecanoic acid, 15:0 (android)	0.25	0.20, 0.28	0.26	0.21, 0.32	0.23	0.21, 0.28	*.210*	*.124*
Pentadecanoic acid, 15:0 (gynoid)	0.23	0.21, 0.26	0.25	0.19, 0.28	0.24	0.21, 0.26	*.851*	
Palmitic acid, 16:0 (android)	22.5	21.2, 24.0	23.2	21.2, 25.8	23.0	22.3, 24.8	*.391*	*.910*
Palmitic acid, 16:0 (gynoid)	20.0	18.5, 22.1	20.6	19.1, 21.9	20.7	18.9, 21.9	*.813*	
Heptadecanoic acid, 17:0 (android)	0.16	0.14, 0.18	0.16	0.12, 0.19	0.15	0.13, 0.19	*.833*	*.519*
Heptadecanoic acid, 17:0 (gynoid)	0.12	0.10, 0.14	0.12	0.06, 0.13	0.11	0.10, 0.14	*.766*	
Stearic acid, 18:0 (android)	2.95	2.54, 3.31	2.99	2.76, 3.33	3.20	2.53, 3.70	*.490*	*.748*
Stearic acid, 18:0 (gynoid)	1.97	1.55, 2.26	2.00	1.82, 2.33	2.00	1.84, 2.33	*.675*	
**SFA (android)**	**28.5**	26.7, 31.6	**29.9**	27.7, 32.5	**29.8**	28.5, 31.9	***.315***	***.851***
**SFA (gynoid)**	**25.1**	22.7, 27.4	**25.5**	23.5, 26.8	**25.6**	23.4, 27.6	***.819***	
Myristoleic acid, 14:1n-5 (android)	0.33	0.22, 0.39	0.30	0.25, 0.35	0.29	0.20, 0.40	*.606*	*.946*
Myristoleic acid, 14:1n-5 (gynoid)	0.35	0.30, 0.47	0.37	0.26, 0.48	0.35	0.29, 0.50	*,962*	
Pentadecenoic acid, 15:1 (android)	0.05	0.00, 0.07	0.00	0.00, 0.06	0.00	0.00, 0.06	*.610*	*.104*
Pentadecenoic acid, 15:1 (gynoid)	0.05	0.00, 0.06	0.02	0.00, 0.07	0.04	0.00, 0.06	*.844*	
Palmitoleic acid, 16:1n-7 (android)	5.16	4.47, 6.19	5.20	4.84, 5.68	4.90	4.05, 5.82	*.624*	*.487*
Palmitoleic acid, 16:1n-7 (gynoid)	7.19	6.15, 8.30	7.60	6.33, 8.83	6.83	6.06, 8.03	*.458*	
Elaidic acid, 18:1n-9t (android)	0.00	0.00, 0.38	0.00	0.00, 0.35	0.00	0.00, 0.29	*.726*	*.332*
Elaidic acid, 18:1n-9t (gynoid)	0.00	0.00, 0.28	0.00	0.00, 0.34	0.00	0.00, 0.35	*.750*	
Oleic acid, 18:1n-9c (android)	50.6	48.7, 51.7	48.6	46.9, 51.2	48.8	47.8, 50.4	*.068*	*.987*
Oleic acid, 18:1n-9c (gynoid)	52.2	49.7, 52.9	50.5	48.2, 53.4	50.9	49.2, 53.0	*.434*	
Cis-vaccenic acid, 18:1n-7 (android)	2.53	2.31, 2.85	2.46	2.23, 2.67	2.43	2.19, 2.68	*.423*	*.692*
Cis-vaccenic acid, 18:1n-7 (gynoid)	2.68	2.38, 2.96	2.69	2.48, 2.88	2.65	2.39, 2.92	*.941*	
Eicosenoic acid, 20:1n-9 (android)	0.41	0.31, 0.50	0.42	0.30, 0.49	0.42	0.30, 0.55	*.872*	*.639*
Eicosenoic acid, 20:1n-9 (gynoid)	0.39	0.30, 0.46	0.36	0.26, 0.44	0.38	0.27, 0.48	*.675*	
Unknown FA1 (android)	0.73	0.65, 0.78	0.70	0.63, 0.76	0.69	0.64, 0.75	*.656*	*.645*
Unknown FA1 (gynoid)	0.86	0.78, 1.00	0.86	0.78, 0.94	0.88	0.75, 0.94	*.798*	
Unknown FA2 (android)	0.14	0.11, 0.15	0.13	0.10, 0.17	0.12	0.09, 0.16	*.678*	*.854*
Unknown FA2 (gynoid)	0.14	0.12, 0.15	0.12	0.06, 0.16	0.12	0.10, 0.15	*.837*	
**MUFA (android)**	**60.1**	58.3, 62.4	**57.9**	56.7, 60.3	**58.3**	56.9, 60.4	***.036*** ^***c***^	***.736***
**MUFA (gynoid)**	**63.4**	61.4, 66.4	**62.2**	61.0, 64.5	**62.8**	60.6, 65.4	***.374***	
Linoleic acid, 18:2n-6 (android)	9.73	8.76, 10.6	10.2	8.95, 11.6	10.4	8.68, 11.5	*.296*	*.689*
Linoleic acid, 18:2n-6 (gynoid)	9.99	8.93, 11.5	10.6	8.93, 12.0	10.2	9.15, 11.5	*.800*	
Linolenic acid (ALA), 18:3n-3 (android)	0.56	0.44, 0.67	0.59	0.45, 0.73	0.55	0.39, 0.77	*.963*	*.237*
Linolenic acid (ALA), 18:3n-3 (gynoid)	0.59	0.44, 0.74	0.57	0.42, 0.72	0.56	0.39, 0.84	*.883*	
Stearidonic acid, 18:4n-3 (android)	0.00	0.00, 0.09	0.00	0.00, 0.00	0.00	0.00, 0.08	*.461*	*.511*
Stearidonic acid, 18:4n-3 (gynoid)	0.00	0.00, 0.09	0.00	0.00, 0.10	0.00	0.00, 0.09	*.930*	
Eicosadienoic acid, 20:2n-6 (android)	0.00	0.00, 0.14	0.00	0.00, 0.09	0.06	0.00, 0.13	*.398*	*.609*
Eicosadienoic acid, 20:2n-6 (gynoid)	0.00	0.00, 0.08	0.00	0.00, 0.08	0.00	0.00, 0.08	*.921*	
Eicosatrienoic acid, 20:3n-6 (android)	0.14	0.00, 0.19	0.09	0.00, 0.15	0.12	0.00, 0.16	*.576*	*.269*
Eicosatrienoic acid, 20:3n-6 (gynoid)	0.15	0.00, 0.18	0.08	0.00, 0.16	0.13	0.00, 0.20	*.466*	
Arachidonic acid, 20:4n-6 (android)	0.20	0.13, 0.33	0.22	0.19, 0.27	0.22	0.14, 0.29	*.841*	*.512*
Arachidonic acid, 20:4n-6 (gynoid)	0.21	0.15, 0.38	0.22	0.18, 0.30	0.21	0.15, 0.33	*.913*	
Docosapentaenoic acid (DPA), 22:5n-3 (android)	0.00	0.00, 0.08	0.00	0.00, 0.11	0.00	0.00, 0.12	*.319*	*.462*
Docosapentaenoic acid (DPA), 22:5n-3 (gynoid)	0.00	0.00, 0.08	0.00	0.00, 0.12	0.00	0.00, 0.12	*.307*	
Docosahexaenoic acid (DHA), 22:6n-3 (android)	0.00	0.00, 0.00	0.00	0.00, 0.09	0.00	0.00, 0.08	*.096*	*.844*
Docosahexaenoic acid (DHA), 22:6n-3 (gynoid)	0.00	0.00, 0.00	0.00	0.00, 0.08	0.00	0.00, 0.03	*.392*	
**PUFA (android)**	**10.7**	9.75, 12.1	**11.9**	9.83, 13.2	**11.5**	9.72, 13.1	***.345***	***.772***
**PUFA (gynoid)**	**11.4**	9.81, 12.8	**12.3**	9.45, 13.2	**11.6**	10.3, 13.0	***.861***	

^*a*^P represents uncorrected genotype difference within each depot.

^*b*^P represents uncorrected genotype difference between android and gynoid depots.

^*c*^MUFA in the android depot: Bonferroni corrected p-values of pairwise genotype comparisons were 0.057 for TT vs AT, 1.000 for AT vs AA and 0.106 for TT vs AA.

**Table 3:** Effect of genotype on fatty acid composition (weight %) of android and gynoid subcutaneous adipose tissue in females and males.

	Females n = 66 (android), n = 67 (gynoid)							Males n = 27 (android), n = 26 (gynoid)						
	TT		AT		AA			TT		AT		AA		
	n = 25 (android), n=27 (gynoid)		n=19 (android, gynoid)		n = 22 (android), n=21 (gynoid)			n=6 (android, gynoid)		n = 12 (android), n=11 (gynoid)		n=9 (android, gynoid)		
	Median weight %	25^th^, 75^th^ percentiles	Median weight %	25^th^, 75^th^ percentiles	Median weight %	25^th^, 75^th^ percentiles	P^*a*^	Median weight %	25^th^, 75^th^ percentiles	Median weight %	25^th^, 75^th^ percentiles	Median weight %	25^th^, 75^th^ percentiles	P^*a*^
Lauric acid, 12:0 (android)	0.29	0.00, 0.43	0.00	0.00, 0.36	0.29	0.00, 0.41	*.695*	0.00	0.00, 0.71	0.14	0.00, 0.41	0.65	0.00, 1.80	*.218*
Lauric acid, 12:0 (gynoid)	0.22	0.00, 0.41	0.00	0.00, 0.30	0.00	0.00, 0.31	*.354*	0.00	0.00, 0.13	0.00	0.00, 0.46	0.36	0.00, 1.55	*.117*
Myristic acid, 14:0 (android)	2.53	2.27, 2.92	2.68	2.38, 3.00	2.61	2.41, 2.83	*.676*	2.89	2.51, 3.13	2.73	2.47, 3.03	2.73	2.20, 3.21	*.649*
Myristic acid, 14:0 (gynoid)	2.18	2.01, 2.59	2.34	2.03, 2.68	2.29	1.89, 2.53	*.809*	2.44	2.15, 2.66	2.26	1.98, 2.80	2.25	1.85, 2.73	*.842*
Pentadecanoic acid, 15:0 (android)	0.24	0.20, 0.26	0.26	0.20, 0.31	0.23	0.21, 0.28	*.574*	0.28	0.23, 0.30	0.26	0.23, 0.33	0.22	0.19, 0.28	*.235*
Pentadecanoic acid, 15:0 (gynoid)	0.23	0.20, 0.26	0.25	0.19, 0.29	0.24	0.22, 0.29	*.604*	0.25	0.21, 0.29	0.23	0.18, 0.28	0.24	0.17, 0.25	*.582*
Palmitic acid, 16:0 (android)	22.0	20.8, 23.4	22.9	21.2, 25.8	23.0	22.1, 23.8	*.392*	24.4	22.5, 25.3	23.7	20.9, 26.2	24.8	22.6, 26.7	*.782*
Palmitic acid, 16:0 (gynoid)	19.9	18.4, 20.9	20.1	19.2, 21.4	20.6	18.2, 21.7	*.732*	21.8	20.3, 23.6	20.7	18.9, 24.0	21.8	19.3, 23.6	*.783*
Heptadecanoic acid, 17:0 (android)	0.16	0.14, 0.18	0.16	0.11, 0.18	0.15	0.13, 0.19	*.980*	0.16	0.13, 0.17	0.18	0.14, 0.19	0.15	0.12, 0.20	*.474*
Heptadecanoic acid, 17:0 (gynoid)	0.12	0.10, 0.14	0.12	0.08, 0.13	0.11	0.10, 0.14	*.879*	0.13	0.11, 0.14	0.12	0.00, 0.13	0.11	0.09, 0.17	*.694*
Stearic acid, 18:0 (android)	3.14	2.56, 3.46	2.98	2.68, 3.28	3.20	2.49, 3.65	*.642*	2.76	2.23, 3.04	3.19	2.77, 3.82	3.33	2.40, 3.78	*.344*
Stearic acid, 18:0 (gynoid)	1.97	1.54, 2.26	1.95	1.81, 2.21	2.02	1.82, 2.34	*.687*	1.98	1.67, 2.31	2.09	1.83, 2.40	1.98	1.56, 2.43	*.880*
**SFA (android)**	**27.8**	26.3, 30.6	**29.5**	27.0, 31,8	**29.4**	28.5, 30.9	***.442***	**31.0**	28.0, 31.6	**30.3**	28.2, 33.3	**32.3**	29.2, 33.4	***.594***
**SFA (gynoid)**	**24.2**	22.2, 27.3	**25.4**	22.7, 26.6	**25.6**	22.6, 26.1	***.827***	**26.4**	24.9, 28.7	**26.1**	23.8, 29.7	**26.5**	23.5, 30.5	***.919***
Myristoleic acid, 14:1n-5 (android)	0.29	0.22, 0.38	0.28	0.24, 0.34	0.28	0.22, 0.42	*.868*	0.35	0.29, 0.45	0.31	0.25, 0.43	0.21	0.18, 0.32	*.074*
Myristoleic acid, 14:1n-5 (gynoid)	0.35	0.29, 0.45	0.34	0.25, 0.47	0.35	0.29, 0.52	*.845*	0.41	0.29, 0.52	0.46	0.34, 0.49	0.37	0.24, 0.48	*.548*
Pentadecenoic acid, 15:1 (android)	0.05	0.00, 0.07	0.00	0.00, 0.06	0.01	0.00, 0.06	*.525*	0.03	0.00, 0.06	0.05	0.00, 0.06	0.00	0.00, 0.05	*.548*
Pentadecenoic acid, 15:1 (gynoid)	0.00	0.00, 0.06	0.04	0.00, 0.07	0.05	0.00, 0.06	*.542*	0.05	0.00, 0.07	0.00	0.00, 0.09	0.00	0.00, 0.07	*.799*
Palmitoleic acid, 16:1n-7 (android)	4.97	4.42, 6.26	5.11	4.72, 5.30	4.95	4.45, 5.78	*.995*	5.53	4.83, 6.35	5.55	4.97, 6.61	4.00	3.60, 6.06	*.317*
Palmitoleic acid, 16:1n-7 (gynoid)	7.19	6.18, 8.46	7.05	6.01, 7.70	6.89	6.26, 7.86	*.873*	7.50	5.81, 8.19	8.79	7.47, 9.49	6.28	5.23, 8.41	*.085*
Elaidic acid, 18:1n-9t (android)	0.00	0.00, 0.42	0.00	0.00, 0.39	0.00	0.00, 0.25	*.540*	0.12	0.00, 0.37	0.00	0.00, 0.33	0.29	0.00, 0.36	*.858*
Elaidic acid, 18:1n-9t (gynoid)	0.00	0.00, 0.12	0.00	0.00, 0.41	0.00	0.00, 0.34	*.129*	0.37	0.22, 1.38	0.00	0.00, 0.26	0.34	0.00, 0.36	*.015* ^*b*^
Oleic acid, 18:1n-9c (android)	50.6	48.9, 51.8	48.8	46.9, 51.5	48.8	47.6, 51.8	*.215*	49.4	48.6, 52.3	47.7	46.5, 51.1	48.9	47.7, 49.8	*.353*
Oleic acid, 18:1n-9c (gynoid)	52.3	49.3, 53.0	50.6	48.7, 53.7	50.8	49.0, 53.4	*.608*	51.1	49.6, 52.9	49.1	47.9, 53.3	51.0	48.3, 51.9	*.781*
Cis-vaccenic acid, 18:1n-7 (android)	2.53	2.13, 2.93	2.49	2.23, 2.67	2.50	2.27, 2.70	*.647*	2.51	2.26, 2.71	2.39	2.22, 2.69	2.18	1.96, 2.74	*.387*
Cis-vaccenic acid, 18:1n-7 (gynoid)	2.66	2.37, 3.21	2.71	2.42, 2.92	2.66	2.51, 2.85	*1.000*	2.69	2.53, 2.86	2.63	2.50, 2.87	2.55	2.09, 2.99	*.901*
Eicosenoic acid, 20:1n-9 (android)	0.46	0.35, 0.58	0.44	0.32, 0.52	0.45	0.32, 0.55	*.773*	0.31	0.28, 0.35	0.35	0.29, 0.47	0.24	0.15, 0.56	*.390*
Eicosenoic acid, 20:1n-9 (gynoid)	0.40	0.33, 0.47	0.40	0.32, 0.45	0.43	0.28, 0.49	*.832*	0.30	0.26, 0.35	0.28	0.24, 0.41	0.28	0.13, 0.47	*.983*
Unknown FA1 (android)	0.75	0.66, 0.78	0.70	0.63, 0.83	0.70	0.65, 0.79	*.669*	0.67	0.61, 0.71	0.69	0.60, 0.70	0.68	0.54, 0.76	*.978*
Unknown FA1 (gynoid)	0.87	0.80, 1.02	0.85	0.79, 1.00	0.90	0.77, 0.96	*.740*	0.77	0.73, 0.88	0.89	0.66, 0.92	0.76	0.64, 0.91	*.656*
Unknown FA2 (android)	0.14	0.11, 0.16	0.13	0.00, 0.14	0.13	0.11, 0.15	*.277*	0.15	0.11, 0,16	0.14	0.12, 0.17	0.09	0.09, 0.16	*.251*
Unknown FA2 (gynoid)	0.13	0.11, 0.15	0.12	0.08, 0.15	0.13	0.10, 0.15	*.693*	0.15	0.12, 0.17	0.15	0.00, 0.17	0.10	0.09, 0.17	*.651*
**MUFA (android)**	**60.7**	57.8, 62.4	**58.5**	56.5, 60.8	**58.9**	56.9, 61.6	***.177***	**59.3**	58.5, 61.2	**57.1**	56.7, 59.8	**57.1**	56.1, 58.6	***.097***
**MUFA (gynoid)**	**63.5**	61.8, 66.5	**62.1**	61.0, 63.7	**63.1**	61.2, 65.7	***.362***	**63.1**	60.8, 66.2	**62.5**	60.4, 67.5	**61.4**	59.1, 64.2	***.555***
Linoleic acid, 18:2n-6 (android)	9.93	8.83, 10.7	10.1	9.51, 11.6	10.4	8.67, 11.6	*.425*	8.91	8.06, 10.3	10.5	8.02, 11.8	10.3	8.85, 11.2	*.646*
Linoleic acid, 18:2n-6 (gynoid)	10.4	9.24, 11.6	11.2	9.65, 12.3	10.4	9.31, 11.6	*.486*	8.59	8.17, 9.93	9.05	8.13, 11.5	10.0	9.13, 11.4	*.294*
Linolenic acid (ALA), 18:3n-3 (android)	0.59	0.48, 0.70	0.60	0.42, 0.75	0.67	0.45, 0.78	*.898*	0.43	0.39, 0.54	0.57	0.45, 0.70	0.42	0.32, 0.77	*.289*
Linolenic acid (ALA), 18:3n-3 (gynoid)	0.63	0.46, 0.78	0.60	0.46, 0.76	0.63	0.42, 0.81	*.934*	0.44	0.36, 0.54	0.46	0.38, 0.67	0.52	0.34, 0.85	*.831*
Stearidonic acid, 18:4n-3 (android)	0.00	0.00, 0.10	0.00	0.00, 0.00	0.00	0.00, 0.09	*.220*	0.00	0.00, 0.08	0.00	0.00, 0.07	0.00	0.00, 0.00	*.514*
Stearidonic acid, 18:4n-3 (gynoid)	0.00	0.00, 0.09	0.00	0.00, 0.10	0.00	0.00, 0.10	*.857*	0.00	0.00, 0.09	0.00	0.00, 0.09	0.00	0.00, 0.04	*.904*
Eicosadienoic acid, 20:2n-6 (android)	0.07	0.00, 0.14	0.00	0.00, 0.09	0.09	0.00, 0.14	*.268*	0.00	0.00, 0.01	0.00	0.00, 0.08	0.00	0.00, 0.06	*.494*
Eicosadienoic acid, 20:2n-6 (gynoid)	0.00	0.00, 0.09	0.00	0.00, 0.09	0.00	0.00, 0.09	*.831*	0.00	0.00, 0.06	0.00	0.00, 0.06	0.00	0.00, 0.09	*.502*
Eicosatrienoic acid, 20:3n-6 (android)	0.17	0.04, 0.19	0.08	0.00, 0.18	0.13	0.07, 0.16	*.215*	0.00	0.00, 0.02	0.12	0.00, 0.15	0.00	0.00, 0.10	*.070*
Eicosatrienoic acid, 20:3n-6 (gynoid)	0.16	0.07, 0.18	0.12	0.00, 0.19	0.15	0.06, 0.21	*.701*	0.01	0.00, 0.07	0.00	0.00, 0.09	0.07	0.00, 0.12	*.725*
Arachidonic acid, 20:4n-6 (android)	0.27	0.13, 0.39	0.22	0.15, 0.33	0.23	0.16, 0.30	*.868*	0.14	0.09, 0,16	0.22	0.19, 0.26	0.16	0.10, 0.28	*.011* ^*c*^
Arachidonic acid, 20:4n-6 (gynoid)	0.23	0.17, 0.39	0.21	0.18, 0.31	0.24	0.16, 0.34	*.663*	0.15	0.09, 0.18	0.25	0.17, 0.29	0.15	0.14, 0.26	*.108*
Docosapentaenoic acid (DPA), 22:5n-3 (android)	0.00	0.00, 0.10	0.00	0.00, 0,11	0.08	0.00, 0.13	*.449*	0.00	0.00, 0.00	0.04	0.00, 0.13	0.00	0.00, 0.07	*.101*
Docosapentaenoic acid (DPA), 22:5n-3 (gynoid)	0.00	0.00, 0.09	0.07	0.00, 0.12	0.00	0.00, 0.12	*.316*	0.00	0.00, 0.02	0.00	0.00, 0.09	0.00	0.00, 0.10	*.643*
Docosahexaenoic acid (DHA), 22:6n-3 (android)	0.00	0.00, 0.00	0.00	0.00, 0.09	0.00	0.00, 0.09	*.317*	0.00	0.00, 0.00	0.00	0.00, 0.11	0.00	0.00, 0.08	*.235*
Docosahexaenoic acid (DHA), 22:6n-3 (gynoid)	0.00	0.00, 0.00	0.00	0.00, 0.07	0.00	0.00, 0.03	*.587*	0.00	0.00, 0.00	0.00	0.00, 0.11	0.00	0.00, 0.08	*.415*
**PUFA (android)**	**11.1**	10.1, 12.2	**11.9**	9.99, 13.6	**11.9**	9.77, 13.2	***.499***	**9.64**	8.62, 11.0	**11.9**	9.25, 12.9	**11.4**	9.31, 12.8	***.386***
**PUFA (gynoid)**	**11.6**	10.4, 12.8	**12.3**	11.1, 13.9	**11.7**	10.4, 13.0	***.555***	**9.21**	8.81, 10.7	**9.77**	9.08, 12.6	**10.6**	9.78, 12.7	***.249***

^*a*^P represents uncorrected genotype difference.

^*b*^Elaidic acid (18:1n-9t) gynoid, males: Bonferroni corrected p-values of pairwise genotype comparisons were 0.012 for TT vs AT, 0.498 for AT vs AA and 0.335 for TT vs AA.

^*c*^Arachidonic acid (20:4n-6) android, males: Bonferroni corrected p-values of pairwise genotype comparisons were 0.010 for TT vs AT, 0.264 for AT vs AA and 0.520 for TT vs AA.

To summarize, our main finding in this section was that the android depot had an overall significant difference between the genotypes in terms of weight % MUFA, when looking at all participants together.

### Effect of depot on FA composition (weight %)

**Fig 2:**
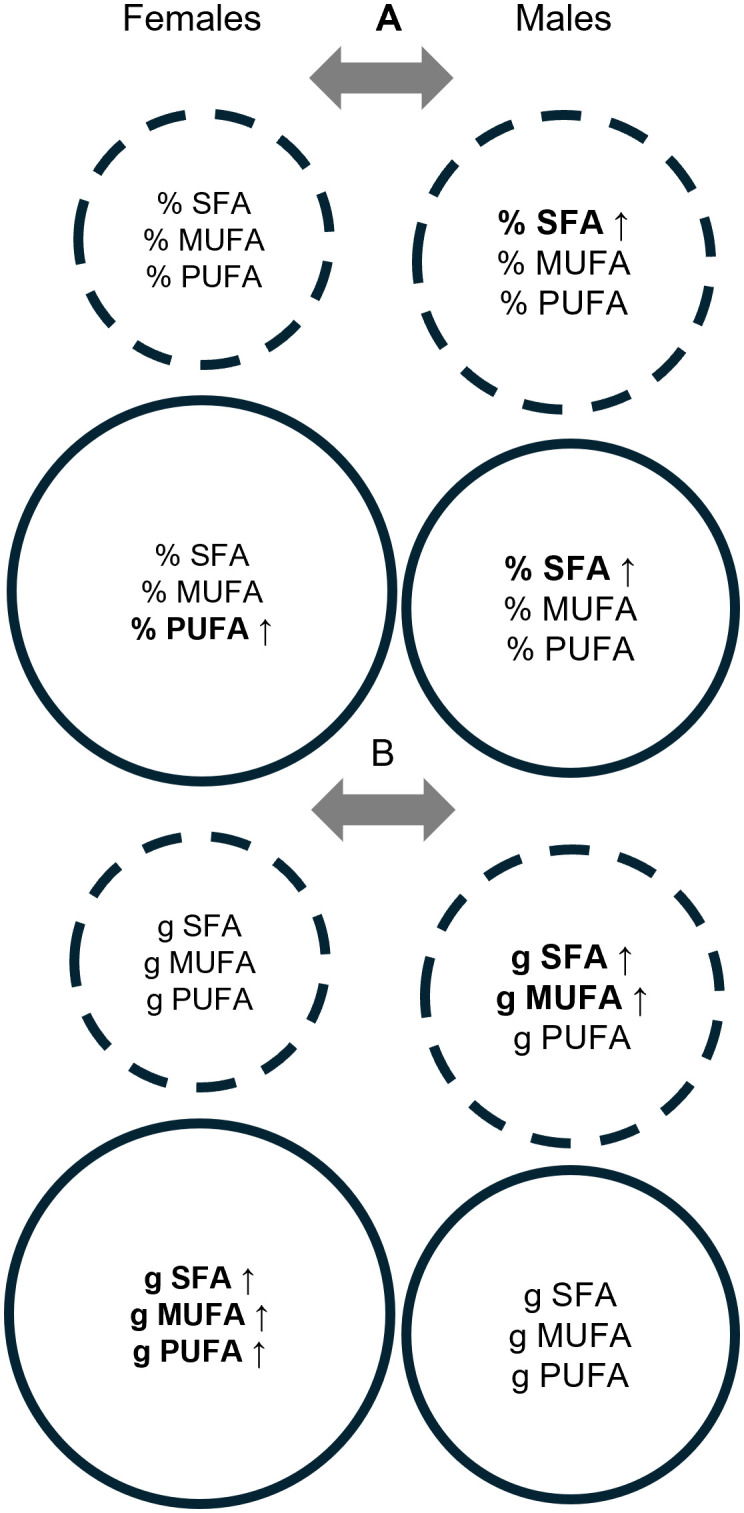
A comparison of fatty acid composition in females and males with obesity. SFA, MUFA and PUFA are shown as proportions in Panel A, weight % and mass content in Panel B, g, in subcutaneous adipose tissue in android (dotted outline) and gynoid depots (solid outline). The bidirectional arrows indicate the comparions between sexes and the areas of the circles are proportional to the depot mass. Males with obesity have significantly more android and less gynoid fat than women. A FA group in bold script is significantly greater than the same FA group in the same depot in the other sex (values and p-values are given in S3 and S5 Tables).

Independent of genotype, the android samples displayed a higher proportion of SFA than the gynoid for every FA except for lauric acid (12:0), and the gynoid depot contained more MUFA for six out of the nine FAs (S2 Table). The proportion of the PUFA linoleic acid (18:2n-6) was significantly higher in the gynoid than in the android depot (S2 Table). Furthermore, males had a higher proportion of SFAs than females in both depots (**Fig 2**, Panel A**).** This was due to the males’ higher presence of palmitic acid (16:0) (S3 Table). MUFAs were mostly similar between sexes (Fig 2, Panel A), although eicosenoic acid (20:1n-9) was higher in females for both depots (S3 Table). The gynoid region PUFAs were significantly higher among females than males (Fig 2, Panel A), due to higher proportions of linoleic (18:2n-6), linolenic (18:3n-3) and eicosatrienoic acid (20:3n-6), while differences between females and males in the android depot were limited to minor FAs (S3 Table).

To summarize the results in this section, for all participants, the android depot consistently contained a greater proportion of SFA (expressed as weight %), than the gynoid depot, a lower proportion of MUFA and a lower proportion of linoleic acid (18:2n-6). There were significant sex differences with higher SFA proportions in both depots in males compared with females, and higher PUFA proportions in the gynoid depot in females, compared with males.

### Effect of genotype on FA mass content (g per depot)

Because we found genotype differences in visceral and gynoid fat mass in males (Table 1), we sought to determine whether this could impact the total depot mass content of specific FAs. In the cohort overall, no significant effect of genotype was found on the mass content of specific or grouped FAs for each depot, or for the calculated difference between depots (**Table 4**). Exploring each sex separately, we found no genotype differences for the total mass of single and grouped FAs within either fat mass depot in females (**Table 5**). In males, the total MUFAs was significantly affected by genotype overall in the gynoid depot (Table 5); however, the effect was no longer significant in Bonferroni-corrected pairwise comparisons (Table 5, footnote *e*). For individual MUFAs we found significantly larger masses of elaidic (18:1n-9t) and oleic (18:1n-9c) acids in the non-risk TT compared with the AT genotype, and a larger mass of cis-vaccenic acid (18:1n-7) in the TT compared with the AA genotype (Table 5, footnotes *b*, *c*, *d*).

**Table 4:** Effect of genotype on fatty acid mass content (g per depot) of android and gynoid subcutaneous adipose tissue.

Fatty acids in adipose tissue subcutaneous depots	TT n=30 (android) n=32 (gynoid)		AT n=31 (android) n=30 (gynoid)		AA n=31 (android) n=30 (gynoid)		Within each depot	Between depots
	Females n = 24 (android), n=26 (gynoid)		Females n = 19 (android, gynoid)		Females n = 22 (android), n=21 (gynoid)			
	Males n = 6 (android, gynoid)		Males n = 12 (android), n=11 (gynoid)		Males n = 9 (android, gynoid)			
	Median weight %	25^th^, 75^th^ percentiles	Median weight %	25^th^, 75^th^ percentiles	Median weight %	25^th^, 75^th^ percentiles	P^*a*^	P^*b*^
Lauric acid, 12:0 (android)	8	0, 19	0	0, 15	12	0, 21	*.451*	*.290*
Lauric acid, 12:0 (gynoid)	0	0, 32	0	0, 28	0	0, 32	*.657*	
Myristic acid, 14:0 (android)	108	83, 158	103	79, 137	106	78, 132	*.641*	*.457*
Myristic acid, 14:0 (gynoid)	198	148, 228	180	129, 224	162	119, 231	*.398*	
Pentadecanoic acid, 15:0 (android)	11	7, 15	10	7, 13	10	7, 13	*.718*	*.275*
Pentadecanoic acid, 15:0 (gynoid)	19	16, 24	18	13, 24	16	13, 26	*.623*	
Palmitic acid, 16:0 (android)	1005	799, 1255	913	669, 1176	948	742, 1106	*.684*	*.496*
Palmitic acid, 16:0 (gynoid)	1740	1418, 2015	1574	1202, 1808	1472	1215, 2012	*.394*	
Heptadecanoic acid, 17:0 (android)	7	5, 9	6	4, 8	7	4, 8	*.702*	*.254*
Heptadecanoic acid, 17:0 (gynoid)	10	7, 13	9	3, 11	9	6, 11	*.343*	
Stearic acid, 18:0 (android)	131	100, 152	109	88, 148	133	95, 157	*.572*	*.685*
Stearic acid, 18:0 (gynoid)	148	126, 197	154	120, 188	146	124, 196	*.920*	
**SFA (android)**	**1273**	994, 1598	**1158**	845, 1490	**1180**	944, 1453	***.659***	***.468***
**SFA (gynoid)**	**2121**	1754, 2452	**1974**	1514, 2266	**1850**	1551, 2538	***.421***	
Myristoleic acid, 14:1n-5 (android)	14	8, 20	12	8, 15	10	8, 16	*.589*	*.495*
Myristoleic acid, 14:1n-5 (gynoid)	29	22, 43	27	18, 42	23	18, 44	*.556*	
Pentadecenoic acid, 15:1 (android)	1	0, 3	0	0, 2	0	0, 2	*.662*	*.640*
Pentadecenoic acid, 15:1 (gynoid)	2	0, 5	1	0, 5	2	0, 5	*.946*	
Palmitoleic acid, 16:1n-7 (android)	239	135, 316	204	141, 276	195	150, 273	*.632*	*.530*
Palmitoleic acid, 16:1n-7 (gynoid)	634	436, 794	589	410, 740	501	392, 785	*.546*	
Elaidic acid, 18:1n-9t (android)	0	0, 20	0	0, 15	0	0, 12	*.761*	*.356*
Elaidic acid, 18:1n-9t (gynoid)	0	0, 25	0	0, 27	0	0, 26	*.893*	
Oleic acid, 18:1n-9c (android)	2199	1684, 2661	1815	1342, 2390	2060	1646, 2417	*.371*	*.347*
Oleic acid, 18:1n-9c (gynoid)	4405	3851, 5040	3756	2914, 4947	3753	3071, 5239	*.209*	
Cis-vaccenic acid, 18:1n-7 (android)	114	78, 147	85	73, 131	98	78, 119	*.342*	*.334*
Cis-vaccenic acid, 18:1n-7 (gynoid)	240	175, 279	188	160, 247	193	162, 253	*.307*	
Eicosenoic acid, 20:1n-9 (android)	19	13, 22	15	10, 20	15	11, 24	*.490*	*.222*
Eicosenoic acid, 20:1n-9 (gynoid)	32	22, 36	26	18, 34	30	18, 39	*.262*	
Unknown FA1 (android)	31	23, 37	25	19, 35	29	24, 35	*.469*	*.163*
Unknown FA1 (gynoid)	72	57, 87	61	45, 84	63	53, 82	*.313*	
Unknown FA2 (android)	5	5, 8	5	3, 6	5	4, 7	*.206*	*.372*
Unknown FA2 (gynoid)	11	8, 14	9	4, 12	10	6, 13	*.248*	
**MUFA (android)**	**2636**	1924, 3237	**2076**	1578, 2935	**2469**	1944, 2783	***.387***	***.325***
**MUFA (gynoid)**	**5444**	4696, 6280	**4690**	3664, 6049	**4621**	3726, 6415	***.250***	
Linoleic acid, 18:2n-6 (android)	448	336, 497	373	306, 520	409	368, 482	*.869*	*.433*
Linoleic acid, 18:2n-6 (gynoid)	823	722, 971	753	568, 1016	820	666, 936	*.641*	
Linolenic acid (ALA), 18:3n-3 (android)	24	19, 29	21	16, 29	22	17, 32	*.511*	*.300*
Linolenic acid (ALA), 18:3n-3 (gynoid)	47	38, 54	43	28, 60	48	27, 65	*.521*	
Stearidonic acid, 18:4n-3 (android)	0	0, 4	0	0, 0	0	0, 3	*.365*	*.697*
Stearidonic acid, 18:4n-3 (gynoid)	0	0, 6	0	0, 8	0	0, 6	*.947*	
Eicosadienoic acid, 20:2n-6 (android)	0	0, 5	0	0, 4	3	0, 6	*.315*	*.589*
Eicosadienoic acid, 20:2n-6 (gynoid)	0	0, 6	0	0, 6	0	0, 6	*.970*	
Eicosatrienoic acid, 20:3n-6 (android)	4	0, 8	3	0, 7	4	0, 7	*.691*	*.650*
Eicosatrienoic acid, 20:3n-6 (gynoid)	10	0, 16	6	0, 12	9	0, 15	*.440*	
Arachidonic acid, 20:4n-6 (android)	9	5, 13	8	6, 12	9	5, 14	*.988*	*.246*
Arachidonic acid, 20:4n-6 (gynoid)	16	12, 26	17	13, 21	18	9, 25	*.977*	
Docosapentaenoic acid (DPA), 22:5n-3 (android)	0	0, 3	0	0, 4	0	0, 6	*.342*	*.293*
Docosapentaenoic acid (DPA), 22:5n-3 (gynoid)	0	0, 6	0	0, 9	0	0, 9	*.303*	
Docosahexaenoic acid (DHA), 22:6n-3 (android)	0	0, 0	0	0, 4	0	0, 3	*.117*	*.367*
Docosahexaenoic acid (DHA), 22:6n-3 (gynoid)	0	0, 0	0	0, 6	0	0, 3	*.352*	
**PUFA (android)**	**498**	381, 542	**408**	329, 590	**470**	399, 566	***.819***	***.520***
**PUFA (gynoid)**	**909**	807, 1120	**802**	642, 1111	**911**	736, 1042	***.634***	

^*a*^P represents uncorrected genotype difference within each depot.

^*b*^P represents uncorrected genotype difference between android and gynoid depots.

**Table 5:** Effect of genotype on fatty acid mass content (g per depot) of android and gynoid subcutaneous adipose tissue in females and males.

	Females n = 65 (android), n = 66 (gynoid)							Males n = 27 (android), n = 26 (gynoid)						
	TT		AT		AA			TT		AT		AA		
	n *=* 24 (android), n=26 (gynoid)		n=19 (android, gynoid)		n = 22 (android), n=21 (gynoid)			n=6 (android, gynoid)		n=12 (android), n=11 (gynoid)		n=9 (android, gynoid)		
	Median g	25^th^, 75^th^ percentiles	Median g	25^th^, 75^th^ percentiles	Median g	25^th^, 75^th^ percentiles	P^*a*^	Median g	25^th^, 75^th^ percentiles	Median g	25^th^, 75^th^ percentiles	Median g	25^th^, 75^th^ percentiles	P^*a*^
Lauric acid. 12:0 (android)	10	0, 18	0	0, 14	10	0, 16	*.760*	0	0, 30	6	0, 17	22	0, 83	*.261*
Lauric acid. 12:0 (gynoid)	10	0, 35	0	0, 32	0	0, 24	*.450*	0	0, 7	0	0, 27	20	0, 118	*.117*
Myristic acid. 14:0 (android)	105	82, 133	103	73, 136	102	71, 128	*.880*	174	131, 190	105	81, 152	119	83, 142	*.209*
Myristic acid. 14:0 (gynoid)	180	144, 225	198	148, 242	168	142, 264	*.796*	225	170, 252	140	101, 182	127	94, 165	*.169*
Pentadecanoic acid. 15:0 (android)	10	7, 12	10	6, 12	10	7, 12	*.990*	16	13, 18	11	9, 14	9	7, 14	*.163*
Pentadecanoic acid. 15:0 (gynoid)	19	15, 24	20	16, 26	20	15, 32	*.832*	23	15, 26	14	9, 20	12	9, 16	*.167*
Palmitic acid. 16:0 (android)	923	744, 1078	892	645, 1038	884	715, 1050	*.867*	1430	1198, 1506	969	719, 1340	1106	872, 1396	*.105*
Palmitic acid. 16:0 (gynoid)	1704	1390, 1876	1745	1401, 2202	1532	1269, 2148	*.879*	2048	1452, 2184	1199	898, 1658	1275	943, 1566	*.067*
Heptadecanoic acid. 17:0 (android)	6	5, 9	5	3, 8	6	4, 8	*.721*	9	6, 10	7	5, 9	8	5, 9	*.501*
Heptadecanoic acid. 17:0 (gynoid)	9	7, 12	9	5, 11	9	6, 12	*.905*	12	7, 13	7	0, 10	8	4, 10	*.132*
Stearic acid. 18:0 (android)	128	93, 149	100	87, 147	123	93, 143	*.643*	150	110, 169	126	91, 155	148	104, 193	*.549*
Stearic acid. 18:0 (gynoid)	145	125, 208	164	147, 205	163	130, 225	*.591*	178	127, 191	122	101, 152	137	86, 162	*.177*
**SFA (android)**	**1189**	941, 1368	**1158**	815, 1345	**1137**	917, 1359	***.863***	**1787**	1472, 1915	**1169**	931, 1660	**1441**	1147, 1815	***.170***
**SFA (gynoid)**	**2099**	1737, 2375	**2160**	1752, 2678	**1909**	1572, 2661	***.875***	**2507**	1772, 2641	**1595**	1132, 2065	**1601**	1177, 2127	***.089***
Myristoleic acid. 14:1n-5 (android)	13	8, 17	11	8, 15	11	7, 18	*.670*	21	15, 27	14	9, 21	10	7, 14	.171
Myristoleic acid. 14:1n-5 (gynoid)	28	22, 39	33	17, 42	25	20, 47	*.964*	37	23, 49	24	19, 38	19	15, 25	*.760*
Pentadecenoic acid. 15:1 (android)	1	0, 3	0	0, 2	0	0, 2	*.516*	2	0, 4	2	0, 3	0	0, 2	*.709*
Pentadecenoic acid. 15:1 (gynoid)	0	0, 5	4	0, 5	4	0, 6	*.487*	5	0, 6	0	0, 6	0	0, 3	*.488*
Palmitoleic acid. 16:1n-7 (android)	221	127, 186	192	143, 228	205	148, 277	*.735*	316	264, 384	243	140, 326	186	158, 284	*.325*
Palmitoleic acid. 16:1n-7 (gynoid)	612	434, 791	683	461, 812	579	432, 826	*.942*	645	445, 806	503	352, 628	348	289, 488	*.081*
Elaidic acid. 18:1n-9t (android)	0	0, 19	0	0, 13	0	0, 5	*.575*	7	0, 22	0	0, 16	13	0, 17	*.858*
Elaidic acid. 18:1n-9t (gynoid)	0	0, 7	0	0, 35	0	0, 28	*.152*	33	21, 121	0	0, 14	14	0, 22	*.010* ^*b*^
Oleic acid. 18:1n-9c (android)	2122	1595, 2401	1652	1342, 2310	2055	1595, 2166	*.550*	2956	2416, 3112	2185	1366, 2532	2145	1864, 2641	*.113*
Oleic acid. 18:1n-9c (gynoid)	4401	3816, 5179	4609	3632, 5231	4217	3580, 5458	*.998*	4497	3722, 4996	2936	2246, 3774	2800	2504, 3647	*.033* ^*c*^
Cis-vaccenic acid. 18:1n-7 (android)	111	72, 133	83	73, 122	97	76, 119	*.505*	147	124, 156	124	68, 132	99	79, 140	*.145*
Cis-vaccenic acid. 18:1n-7 (gynoid)	235	172, 282	225	175, 289	205	183, 283	*.939*	242	187, 276	161	116, 196	142	113, 184	*.031* ^*d*^
Eicosenoic acid. 20:1n-9 (android)	19	13, 22	14	11, 16	17	11, 23	*.466*	19	12, 21	16	10, 20	13	7, 26	*.704*
Eicosenoic acid. 20:1n-9 (gynoid)	34	23, 39	29	22, 41	31	23, 44	*.723*	25	21, 31	20	13, 26	13	10, 30	*. 339*
Unknown FA1 (android)	29	22, 35	24	19, 32	29	24, 34	*.564*	38	30, 43	30	20, 37	28	24, 42	*.370*
Unknown FA1 (gynoid)	76	59, 89	70	57, 94	70	61, 89	*.933*	68	54, 77	44	34, 71	42	36, 58	*.071*
Unknown FA2 (android)	5	4, 8	4	0, 6	5	3, 7	*.120*	8	6, 10	6	4, 8	5	4, 6	*.186*
Unknown FA2 (gynoid)	11	8, 14	10	5, 12	11	7, 14	*.534*	13	10, 16	8	0, 13	7	5, 10	*.082*
**MUFA (android)**	**2536**	1848, 2881	**1974**	1578, 2781	**2496**	1873, 2659	***.522***	**3530**	2896, 3780	**2709**	1626, 3025	**2457**	2181, 3116	***.108***
**MUFA (gynoid)**	**5413**	4612, 6296	**5593**	4236, 6553	**4983**	4434, 6513	***.983***	**5613**	4569, 6283	**3748**	2761, 4778	**3322**	2964, 4463	***.044*** ^***e***^
Linoleic acid. 18:2n-6 (android)	432	326, 471	339	300, 520	416	337, 482	*.983*	500	444, 552	383	332, 582	403	387, 631	*.381*
Linoleic acid. 18:2n-6 (gynoid)	889	729, 1054	941	714, 1176	866	773, 1037	*.822*	770	675, 808	520	410, 633	557	454, 807	*.106*
Linolenic acid (ALA). 18:3n-3 (android)	24	18, 30	20	16, 24	24	18, 32	*.367*	24	21, 29	24	16, 31	19	14, 38	*.834*
Linolenic acid (ALA). 18:3n-3 (gynoid)	50	41, 57	45	32, 69	55	39, 67	*.800*	38	32, 41	29	20, 44	27	19, 53	*.683*
Stearidonic acid. 18:4n-3 (android)	0	0, 4	0	0, 0	0	0, 4	*.185*	0	0, 5	0	0, 3	0	0, 0	*.486*
Stearidonic acid. 18:4n-3 (gynoid)	0	0, 6	0	0, 8	0	0, 7	*.797*	0	0, 7	0	0, 5	0	0, 2	*.795*
Eicosadienoic acid. 20:2n-6 (android)	1	0, 5	0	0, 4	3	0, 6	*.174*	0	0, 1	0	0, 5	0	0, 3	*.541*
Eicosadienoic acid. 20:2n-6 (gynoid)	0	0, 7	0	0, 8	0	0, 6	*.809*	0	0, 4	0	0, 3	0	0, 6	*.580*
Eicosatrienoic acid. 20:3n-6 (android)	6	1, 9	3	0, 6	5	3, 7	*.199*	0	0, 1	4	0, 7	0	0, 5	*.102*
Eicosatrienoic acid. 20:3n-6 (gynoid)	12	4, 17	10	0, 15	12	4, 18	*.662*	1	0, 5	0	0, 5	3	0, 8	*.796*
Arachidonic acid. 20:4n-6 (android)	10	5, 15	7	5, 9	9	5, 14	*.722*	8	3, 10	11	7, 13	7	5, 13	*.349*
Arachidonic acid. 20:4n-6 (gynoid)	18	12, 30	17	13, 25	22	13, 28	*.830*	12	7, 14	16	11, 20	10	6, 18	*.641*
Docosapentaenoic acid (DPA). 22:5n-3 (android)	0	0, 4	0	0, 4	2	0, 6	*.390*	0	0, 0	1	0, 6	0	0, 4	*.128*
Docosapentaenoic acid (DPA). 22:5n-3 (gynoid)	0	0, 6	5	0, 10	0	0, 9	*.327*	0	0, 1	0	0, 8	0	0, 6	*.616*
Docosahexaenoic acid (DHA). 22:6n-3 (android)	0	0, 0	0	0, 4	0	0, 3	*.393*	0	0, 0	0	0, 5	0	0, 4	*.235*
Docosahexaenoic acid (DHA). 22:6n-3 (gynoid)	0	0, 0	0	0, 5	0	0, 3	*.543*	0	0, 0	0	0, 9	0	0, 5	*.407*
**PUFA (android)**	**477**	373, 539	**382**	322, 552	**468**	382, 556	***.869***	**536**	467, 600	**442**	362, 632	**470**	419, 666	***.535***
**PUFA (gynoid)**	**974**	809, 1171	**1039**	797, 1356	**968**	843, 1185	***.853***	**817**	733, 862	**610**	452, 766	**673**	474, 853	***.149***

^*a*^P represents uncorrected genotype difference.

^*b*^Elaidic acid (18:1n-9t) gynoid, males: Bonferroni corrected p-values of pairwise genotype comparisons were 0.008 for TT vs AT, 1.000 for AT vs AA and 0.113 for TT vs AA.

^*c*^Oleic acid (18:1n-9c) gynoid, males: Bonferroni corrected p-values of pairwise genotype comparisons were 0.050 for TT vs AT, 1.000 for AT vs AA and 0.062 for TT vs AA.

^*d*^Cis-vaccenic acid (18:1n-7) gynoid, males: Bonferroni corrected p-values of pairwise genotype comparisons were 0.066 for TT vs AT, 1.000 for AT vs AA and 0.044 for TT vs AA.

^*e*^MUFA in the gynoid depot, males: Bonferroni corrected p-values of pairwise genotype comparisons were 0.080 for TT vs AT, 1.000 for AT vs AA and 0.066 for TT vs AA.

To summarize the main results of this section, no genotype effects within each depot or between the depots was found, when looking at all participants together when expressing FA content as g per depot, and no genotype effects on grouped FA mass content in females and males analysed separately.

### Effect of depot on FA mass content (g per depot)

Independent of genotype, the gynoid samples displayed a higher content of most single and grouped FAs than the android when the proportions were corrected for depot mass (S4 Table). Furthermore, there were significant sex differences within each depot. Females had a larger mass of total SFA, MUFA and PUFA than males in the gynoid depot (Fig 2, Panel B), including larger mass of every single SFA except for FAs 12:0 and 17:0, larger mass of the major single MUFAs, and of the four major single PUFAs (18:2n-6, 18:2n-3, 20:3n-6, 20:4n-6) (S5 Table). Males had a larger mass of total SFA and MUFA than females in the android depot (Fig 2, Panel B), including larger mass of every single SFA except for FA 12:0, and of the major MUFA oleic acid (18:1n-9c) (S5 Table). Despite no sex difference in the total PUFA content of the android depot, females had significantly larger mass of the minor FAs 20:2n-6 and 20:3n-6 than males (S5 Table).

To summarize this section, there was a significantly higher content of most FAs (individual and grouped) in the gynoid compared with the android depot when including all participants. The masses of total SFA and MUFA were greater in gynoid than android depots for females, in line with sex differences in body fat distribution. However, for PUFA, a significant difference between depots was only found in females.

## Discussion

We have previously examined a possible association of the *FTO* rs9939609 risk allele with different metabolic parameters in a study population with obesity [15]. We found a risk allele effect on the respiratory quotient, RQ, related to a lower fat oxidation in the fasted state [16]. On the other hand, we found no clear association of the risk allele with self-reported dietary intake including SFA, MUFA and PUFA [17], or with appetite-related hormones [18]. Likewise an exploration of non-esterified fatty acids (NEFA) dynamics gave negative results [19]. In the present study, in the same cohort, we investigated the FA composition and FA content, corrected for depot size, of subcutaneous adipose tissue.

Contrary to our hypothesis, we did not find that *FTO* rs9939609 variant alleles AA, AT and TT were associated with individual FAs, when expressed either as proportion of total fatty acids, or corrected for depot mass, in either the android or gynoid depots. We did find a borderline significance for a higher proportion of total MUFA in the TT compared with the AT genotype in the android depot (Table 2). The reason for this is unclear. It could be related to the role of *FTO* in the regulation of lipolysis, as reviewed by Yang *et al* [5]. Sex affects several parameters of lipid metabolism and body composition [24]; it was therefore important to determine any genotype effect on the FA composition separately, in females and males. In the females recruited to our study, we found no effect of genotype on the FA composition of either adipose tissue depot. This finding differs from a previous study that recruited younger females with a lower BMI [25]. In males we found a higher proportion of elaidic acid (18:1n-9t) and, when corrected for depot size, also of oleic (18:1n-9c) and cis-vaccenic acid (18:1n-7), in the non-risk TT compared with either AT or AA genotypes, in the gynoid depot (Tables 3 and 4). We interpret these findings with caution due to the small numbers of males with the TT genotype.

Independent of genotype, we found significant differences in the proportion of many individual FAs between android and gynoid adipose tissue with more SFA in the android and more MUFA in the gynoid depots (S2 Table). These within-person differences must be explained by metabolic differences rather than diet per se, but we could not detect any genotype-associated modulation of such FA differences (Table 2). When corrected for depot mass, all single FA and groups (also all the SFAs) had a higher mass in the gynoid than the android depot, probably largely reflecting the larger gynoid than android fat mass in this study population with obesity class 2 and 3 (S1 Table). We found a higher proportion of palmitic acid (16:0) and a lower proportion of palmitoleic acid (16:1 n-7) in android compared with gynoid depots (S2 Table). These specific differences in sixteen-carbon FAs have previously been attributed to differences in *de novo* FA synthesis and desaturation [8].

We found higher proportions of linolenic acid (18:3n-3, a precursor for LC n-3 PUFA synthesis) in females than males in the gynoid depot (S3 Table), but no difference in the proportion of DHA (22:6n-3). Others have reported a higher proportion of 22:6n-3 in adipose tissue in females compared with males, as reviewed by Hodson *et al* [7]. Our findings of higher linoleic acid (18:2n-6) in females compared with males in the gynoid depot, and of higher eicosatrienoic acid (20:3n-6) in both depots, have not previously been reported (S3 Table) and could be related to diet or metabolic pathways. The results of the above-mentioned PUFAs were amplified when corrected for depot mass, revealing also a higher amount of arachidonic acid (20:4n-6) in females compared with males in the gynoid mass (S5 Table). It is remarkable that the females had a larger mass of the minor PUFAs eicosadienoic (20:2n-6) and eicosatrienoic (20:3n-6) acids than males in the android depot, even though the males had a larger android subcutaneous adipose tissue depot *per se*, compared with females. Differences in FA composition between sexes could also be due to differences in diet [7]. It would be interesting to relate these sex differences in FA composition to known differences in the health benefits of gynoid versus android subcutaneous adipose tissue [26].

Strengths of this study include the investigation of a relatively large cohort of well-described subjects with obesity. Such a population appears to be rather unique for a study of an *FTO* obesity-risk allele. We purposely did not recruit individuals with overt diabetes in the current study since a co-presence of hyperglycemia would have complicated interpretations of results. The method used for obtaining adipose tissue samples seems optimal since it is well-tolerated by the participants and is easy to perform in the hands of study nurses. Both our approach and consequent measurements appear to be novel. To the best of our knowledge, measurements of FA composition probing *FTO* obesity risk alleles have not previously been reported in any subcutaneous adipose tissue depots. The FA data presented here, apart from genotype analysis, confirm some aspects of FA composition in different subcutaneous adipose tissue depots that have been published by others [7], and this concurrence could strengthen the validity of our results on genotype. Another strength is that we also present FA composition of the gynoid and android depots as mass results, not only as proportions (weight %). This is a new approach which in our view adds a relevant aspect to the interpretation of our results.

Our study also has limitations. It was not population-based. All individuals who could potentially participate in our study were recognized as referrals to a hospital-based obesity clinic, and the study was performed with Norwegian participants. The impact of our findings is influenced by these conditions. Investigations based on a population-based survey of individuals with obesity of different ethnicities would be a relevant complement to the present study. Caution should also be applied to the finding that the TT genotype in males was associated with a smaller visceral fat mass and a larger gynoid depot (Table 1). We acknowledge that the estimation of subcutaneous android fat mass was not direct and was dependent on the subtraction of visceral fat mass from the total android fat mass measured by DXA. Comparisons with the FA composition of a visceral depot would be relevant but was not carried out in this study. Another limitation is the lack of gene expression data of the *FTO* locus in the current study. We acknowledge that advanced age can influence tissue biology and FA composition [27] and may confound the interpretation of obesity-related metabolic findings [28,29]. However, these studies did not investigate the *FTO* locus related to the FA composition of adipose tissue. Their conclusion was that the *FTO* effect on body composition appeared to be weakened with age, which could also have had an impact on our results, even though our participants had a median age of 42 y. The use of a single risk allele probe, here rs9939609, constitutes a theoretical limitation; however, rs9939609 has been employed in multiple other studies and, after searching the literature, we find no evidence that results with rs9939609 are contradictory to results using other probes.

In conclusion, we report generally negative findings on the effects of the *FTO* rs9939609 risk allele on the FA composition of subcutaneous adipose tissue in the cohort as a whole. The novel finding of higher mass of oleic acid in the gynoid depot of males with the non-risk *FTO* TT genotype requires further studies for validation, especially because these results occur in the male genotype group with the fewest number of participants. Correcting the FA proportions by the gynoid and android depot mass, revealed sex differences of subcutaneous adipose tissue depots not seen before. Health implications regarding a proportional presence of individual FAs or FA groups compared with the total mass of the same FAs, and in different subcutaneous adipose tissue depots, should be the subject of thorough research in the field.

## Supporting information

S1 File Inclusivity in global research. (DOCX)

S1 Table Characteristics of participants by sex. (DOCX)

S2 Table Fatty acid composition (weight %) of android and gynoid adipose tissue, all participants. (DOCX)

S3 Table Fatty acid composition (weight %) of android and gynoid adipose tissue in females and males. (DOCX)

S4 Table Fatty acid mass content (g per depot) of android and gynoid adipose tissue, all participants. (DOCX)

S5 Table Fatty acid mass content (g per depot) of android and gynoid adipose tissue in females and males. (DOCX)
