# Supplementary material for: Impact of the FTO rs9939609 risk allele on subcutaneous adipose tissue fatty acid composition in adults with obesity class 2 and 3
Source: PLoS One. 2026 Jun 17;21(6):e0351698. doi: 10.1371/journal.pone.0351698 (PMC13274855; doi:10.1371/journal.pone.0351698)
Supplement: S1 Table — (DOCX) [file pone.0351698.s002.docx]

**S1 Table**. **Characteristics of participants by sex**

|  | **Females** | | **Males** | | **Sex difference** |
| --- | --- | --- | --- | --- | --- |
|  | n=68^a^ | | n=27 | |  |
|  | Median | 25^th^, 75^th^ percentiles | median | 25^th^, 75^th^ percentiles | P-values |
| Age, year, n=95 | 44 | 31, 51 | 41 | 31, 49 | *.795* |
| Height, cm, n=95 | 167 | 163, 171 | 180 | 175, 184 | *<.001* |
| Weight, kg, n=95 | 118.2 | 105.6, 131.7 | 148.1 | 127.1, 155.6 | *<.001* |
| BMI, kg/m^2^, n=95 | 42.0 | 38.2, 46.2 | 44.4 | 40.6, 47.4 | *.184* |
| Visceral fat mass, g, n=94 | 718 | 552, 868 | 924 | 574, 1068 | *.013* |
| Android fat mass, g, n=94 | 3718 | 3124, 4555 | 4985 | 3656, 5788 | *.005* |
| Gynoid fat mass, g, n=94 | 8480 | 6910, 10070 | 6110 | 5350, 8150 | *<.001* |
| Fat mass, kg, n=94 | 45.7 | 39.6, 53.6 | 47.7 | 38.4, 54.1 | *.861* |

^a^For the four fat mass variables, n=67 for females
