# Supplementary material for: Impact of the FTO rs9939609 risk allele on subcutaneous adipose tissue fatty acid composition in adults with obesity class 2 and 3
Source: PLoS One. 2026 Jun 17;21(6):e0351698. doi: 10.1371/journal.pone.0351698 (PMC13274855; doi:10.1371/journal.pone.0351698)
Supplement: S2 Table — (DOCX) [file pone.0351698.s003.docx]

**S2 Table**. **Fatty acid composition (weight %) of android and gynoid adipose tissue, all participants**

|  | **Android FA**, n=93 | | **Gynoid FA**, n=93 | | **Depot difference** | | P-value |
| --- | --- | --- | --- | --- | --- | --- | --- |
|  | (females n=66, males n=27) | | (females n=67, males n=26) | | (gynoid) minus (android), n=91 | |  |
|  | Median weight % | 25^th^, 75^th^ percentiles | Median weight % | 25^th^, 75^th^ percentiles | Median weight % | 25^th^, 75^th^ percentiles |  |
| Lauric acid, 12:0 | 0.26 | 0.00, 0.43 | 0.00 | 0.00, 0.36 | 0.00 | -0.08, 0.00 | *.053* |
| Myristic acid, 14:0 | 2.68 | 2.35, 2.96 | 2.25 | 1.99, 2.61 | -0.35 | -0.51, -0.20 | *< .001* |
| Pentadecanoic acid, 15:0 | 0.25 | 0.21, 0.29 | 0.24 | 0.21, 0.27 | -0,01 | -0.02, 0.01 | *.003* |
| Palmitic acid, 16:0 | 22.96 | 21.4, 25.0 | 20.5 | 18.8, 21.9 | -2.54 | -3.93, -1.80 | *< .001* |
| Heptadecanoic acid, 17:0 | 0.16 | 0.13, 0.18 | 0.12 | 0.10, 0.14 | -0.04 | -0.07, -0.02 | *< .001* |
| Stearic acid, 18:0 | 3.10 | 2.57, 3.43 | 1.99 | 1.67, 2.29 | -1.01 | -1.29, -0.77 | *< .001* |
| **SFA** | **29.5** | 27.5, 31.7 | **24.4** | 23.2, 27.3 | **-4.11** | -5.73, -2.87 | ***< .001*** |
| Myristoleic acid, 14:1n-5 | 0.28 | 0.22, 0.37 | 0.36 | 0.29, 0.47 | 0.07 | 0.02, 0.14 | *< .001* |
| Pentadecenoic acid, 15:1 | 0.00 | 0.00, 0.06 | 0.04 | 0.00, 0.06 | 0.00 | 0.00, 0.00 | *.755* |
| Palmitoleic acid, 16:1n-7 | 5.11 | 4.42, 5.86 | 7.28 | 6.17, 8.41 | 1.93 | 1.43, 2.87 | *< .001* |
| Elaidic acid, 18:1n-9t | 0.00 | 0.00, 0.34 | 0.00 | 0.00, 0.34 | 0.00 | 0.00, 0.01 | *.843* |
| Oleic acid, 18:1n-9c | 49.6 | 47.6, 51.4 | 51.3 | 49.1, 53.1 | 1.53 | 0.92, 2.54 | *< .001* |
| Cis-vaccenic acid, 18:1n-7 | 2.48 | 2.25, 2.72 | 2.68 | 2.41, 2.91 | 0.20 | 0.06, 0.37 | *< .001* |
| Eicosenoic acid, 20:1n-9 | 0.42 | 0.30, 0.52 | 0.38 | 0.28, 0.47 | -0.05 | -0.10, 0.00 | *< .001* |
| Unknown FA1 | 0.70 | 0.64, 0.78 | 0.86 | 0.77, 0.96 | 0.15 | 0.07, 0.22 | *< .001* |
| Unknown FA2 | 0.13 | 0.10, 0.16 | 0.13 | 0.10, 0.15 | -0.01 | -0.02, 0.01 | *.110* |
| **MUFA** | **58.6** | 57.0, 61.3 | **62.7** | 61.0, 65.8 | **4.07** | 2.61, 5.35 | ***< .001*** |
| Linoleic acid, 18:2n-6 | 10.2 | 8.79, 11.4 | 10.3 | 9.04, 11.6 | 0.14 | -0.15, 0.51 | *.020* |
| Linolenic acid (ALA), 18:3n-3 | 0.57 | 0.42, 0.72 | 0.57 | 0.42, 0.75 | -0.01 | -0.06, 0.04 | *.476* |
| Stearidonic acid, 18:4n-3 | 0.00 | 0.00, 0.08 | 0.00 | 0.00, 0.09 | 0.00 | 0.00, 0.03 | *.188* |
| Eicosadienoic acid, 20:2n-6 | 0.00 | 0.00, 0.12 | 0.00 | 0.00, 0.08 | 0.00 | -0.05, 0.00 | *.008* |
| Eicosatrienoic acid, 20:3n-6 | 0.11 | 0.00, 0.18 | 0.12 | 0.00, 0.18 | 0,00 | -0,02, 0.04 | *.501* |
| Arachidonic acid, 20:4n-6 | 0.22 | 0.15, 0.31 | 0.21 | 0.16, 0.33 | 0.00 | -0.04, 0.03 | *.485* |
| Docosapentaenoic acid (DPA), 22:5n-3 | 0.00 | 0.00, 0.11 | 0.00 | 0.00, 0.10 | 0.00 | -0.01, 0.00 | *.440* |
| Docosahexaenoic acid (DHA), 22:6n-3 | 0.00 | 0.00, 0.07 | 0.00 | 0.00, 0.03 | 0.00 | 0.00, 0.00 | *.112* |
| **PUFA** | **11.4** | 9.80, 12.9 | **11.5** | 9.85, 12.9 | **0.04** | -0.38, 0.57 | **.309** |
