# Supplementary material for: Impact of the FTO rs9939609 risk allele on subcutaneous adipose tissue fatty acid composition in adults with obesity class 2 and 3
Source: PLoS One. 2026 Jun 17;21(6):e0351698. doi: 10.1371/journal.pone.0351698 (PMC13274855; doi:10.1371/journal.pone.0351698)
Supplement: S3 Table — (DOCX) [file pone.0351698.s004.docx]

**S3 Table**. **Fatty acid composition (weight %) of android and gynoid adipose tissue in females and males**

|  | **Females** | | **Males** | | Android difference | **Females** | | **Males** | | Gynoid difference |
| --- | --- | --- | --- | --- | --- | --- | --- | --- | --- | --- |
|  | Android, n=66 | | Android, n=27 | |  | Gynoid, n=67 | | Gynoid, n=26 | |  |
|  | Median weight % | 25^th^, 75^th^ percentiles | Median weight % | 25^th^, 75^th^ percentiles | P-value | Median weight % | 25^th^, 75^th^ percentiles | Median weight % | 25^th^, 75^th^ percentiles | P-value |
| Lauric acid, 12:0 | 0.27 | 0.00, 0.42 | 0.26 | 0.00, 0.65 | *.531* | 0.00 | 0.00, 0.34 | 0.00 | 0.00, 0.54 | *.460* |
| Myristic acid, 14:0 | 2.61 | 2.36, 2.96 | 2.78 | 2.33, 3.06 | *.370* | 2.23 | 2.01, 2.59 | 2.33 | 1.96, 2.77 | *.436* |
| Pentadecanoic acid, 15:0 | 0.24 | 0.20, 0.28 | 0.26 | 0.22, 0.30 | *.260* | 0.24 | 0.21, 0.27 | 0.24 | 0.18, 0.27 | *.421* |
| Palmitic acid, 16:0 | 22.7 | 21.2, 24.1 | 24.0 | 22.4, 25.7 | *.022* | 20.0 | 18.5, 21.4 | 21.1 | 19.7, 23.4 | *.024* |
| Heptadecanoic acid, 17:0 | 0.16 | 0.13, 0.18 | 0.16 | 0.14, 0.18 | *.626* | 0.11 | 0.10, 0.14 | 0.12 | 0.09, 0.14 | *.732* |
| Stearic acid, 18:0 | 3.12 | 2.58, 3.34 | 2.95 | 2.55, 3.65 | *.966* | 1.99 | 1.64, 2.28 | 2.03 | 1.81, 2.33 | *.694* |
| **SFA** | **28.9** | 27.0, 31.2 | **30.7** | 28.3, 32.8 | ***.033*** | **25.2** | 22.4, 26.6 | **26.2** | 23.9, 29.3 | ***.049*** |
| Myristoleic acid, 14:1n-5 | 0.28 | 0.22, 0.37 | 0.30 | 0.22, 0.37 | *.783* | 0.35 | 0.29, 0.47 | 0.42 | 0.29, 0.47 | *.373* |
| Pentadecenoic acid, 15:1 | 0.00 | 0.00, 0.06 | 0.02 | 0.00, 0.06 | *.913* | 0.04 | 0.00, 0.06 | 0.00 | 0.00, 0.07 | *.906* |
| Palmitoleic acid, 16:1n-7 | 4.99 | 4.49, 5.76 | 5.24 | 3.85, 6.19 | *.441* | 7.11 | 6.24, 7.92 | 7.99 | 5.83, 8.86 | *.244* |
| Elaidic acid, 18:1n-9t | 0.00 | 0.00, 0.30 | 0.00 | 0.00, 0.36 | *.327* | 0.00 | 0.00, 0.32 | 0.13 | 0.00, 0.35 | *.295* |
| Oleic acid, 18:1n-9c | 49.8 | 47.8, 51.5 | 48.7 | 47.4, 50.9 | *.310* | 51.4 | 49.2, 53.3 | 50.6 | 48.2, 52.5 | *.190* |
| Cis-vaccenic acid, 18:1n-7 | 2.50 | 2.29, 2.77 | 2.43 | 2.18, 2.71 | *.165* | 2.68 | 2.42, 2.92 | 2.66 | 2.36, 2.88 | *.526* |
| Eicosenoic acid, 20:1n-9 | 0.44 | 0.30, 0.55 | 0.31 | 0.24, 0.42 | *.005* | 0.40 | 0.31, 0.47 | 0.28 | 0.24, 0.39 | *.005* |
| Unknown FA1 | 0.72 | 0.65, 0.78 | 0.69 | 0.62, 0.70 | *.034* | 0.87 | 0.79, 0.99 | 0.80 | 0.71, 0.91 | *.038* |
| Unknown FA2 | 0.13 | 0.11, 0.15 | 0.13 | 0.09, 0.17 | *.474* | 0.13 | 0.10, 0.15 | 0.13 | 0.09, 0.16 | *.451* |
| **MUFA** | **59.2** | 56.9, 61.6 | **58.0** | 56.9, 59.5 | ***.130*** | **63.1** | 61.3, 65.9 | **62.6** | 60.3, 65.5 | ***.360*** |
| Linoleic acid, 18:2n-6 | 10.2 | 8.84, 11.3 | 9.73 | 8.61, 11.5 | *.441* | 10.8 | 9.5, 11.7 | 9.12 | 8.4, 11.2 | *.013* |
| Linolenic acid (ALA), 18:3n-3 | 0.60 | 0.47, 0.73 | 0.46 | 0.38, 0.63 | *.055* | 0.61 | 0.46, 0.76 | 0.47 | 0.37, 0.64 | *.042* |
| Stearidonic acid, 18:4n-3 | 0.00 | 0.00, 0.08 | 0.00 | 0.00, 0.00 | *.407* | 0.00 | 0.00, 0.10 | 0.00 | 0.00, 0.08 | *.394* |
| Eicosadienoic acid, 20:2n-6 | 0.07 | 0.00, 0.14 | 0.00 | 0.00, 0.06 | *.012* | 0.00 | 0.00, 0.09 | 0.00 | 0.00, 0.07 | *.298* |
| Eicosatrienoic acid, 20:3n-6 | 0.13 | 0.00, 0.19 | 0.00 | 0.00, 0.13 | *.005* | 0.15 | 0.00, 0.20 | 0.00 | 0.00, 0.09 | *< .001* |
| Arachidonic acid, 20:4n-6 | 0.24 | 0.15, 0.33 | 0.19 | 0.13, 0.23 | *.078* | 0.23 | 0.17, 0.35 | 0.18 | 0.14, 0.26 | *.076* |
| Docosapentaenoic acid (DPA), 22:5n-3 | 0.00 | 0.00, 0.11 | 0.00 | 0.00, 0.09 | *.227* | 0.00 | 0.00, 0.11 | 0.00 | 0.00, 0.09 | *.223* |
| Docosahexaenoic acid (DHA), 22:6n-3 | 0.00 | 0.00, 0.07 | 0.00 | 0.00, 0.08 | *.965* | 0.00 | 0.00, 0.04 | 0.00 | 0.00, 0.00 | *.548* |
| **PUFA** | **11.5** | 9.99, 13.0 | **10.4** | 9.06, 12.8 | ***.190*** | **11.9** | 10.4, 13.1 | **9,79** | 9,08, 12.5 | ***.006*** |
