# Supplementary material for: Impact of the FTO rs9939609 risk allele on subcutaneous adipose tissue fatty acid composition in adults with obesity class 2 and 3
Source: PLoS One. 2026 Jun 17;21(6):e0351698. doi: 10.1371/journal.pone.0351698 (PMC13274855; doi:10.1371/journal.pone.0351698)
Supplement: S4 Table — (DOCX) [file pone.0351698.s005.docx]

**S4 Table**. **Fatty acid mass content (g per depot) of android and gynoid adipose tissue, all participants**

|  | **Android FA**, n=92 | | **Gynoid FA**, n=92 | | **Depot difference** | | P-value |
| --- | --- | --- | --- | --- | --- | --- | --- |
|  | (females n=65, males n=27) | | (females n=66, males n=26) | | (gynoid) minus (android), n=90 | |  |
|  | Median  g | 25^th^, 75^th^ percentiles | Median  g | 25^th^, 75^th^ percentiles | Median  g | 25^th^, 75^th^ percentiles |  |
| Lauric acid, 12:0 | 9 | 0, 17 | 0 | 0, 31 | 0 | 0, 13 | *.035* |
| Myristic acid, 14:0 | 107 | 80, 140 | 171 | 137, 227 | 65 | 39, 105 | *< .001* |
| Pentadecanoic acid, 15:0 | 10 | 7, 13 | 18 | 14, 24 | 8 | 5, 13 | *< .001* |
| Palmitic acid, 16:0 | 957 | 740, 1207 | 1581 | 1245, 1947 | 655 | 359, 930 | *< .001* |
| Heptadecanoic acid, 17:0 | 7 | 5, 8 | 9 | 7, 12 | 3 | 0, 5 | *< .001* |
| Stearic acid, 18:0 | 129 | 91, 150 | 150 | 125, 192 | 35 | 1, 66 | *< .001* |
| **SFA** | **1204** | 940, 1483 | **1982** | 1574, 2423 | **781** | 395, 1140 | ***< .001*** |
| Myristoleic acid, 14:1n-5 | 12 | 8, 17 | 27 | 19, 42 | 17 | 11, 26 | *< .001* |
| Pentadecenoic acid, 15:1 | 0 | 0, 2 | 2 | 0, 5 | 0 | 0, 3 | *< .001* |
| Palmitoleic acid, 16:1n-7 | 207 | 145, 290 | 576 | 424, 753 | 345 | 250, 498 | *< .001* |
| Elaidic acid, 18:1n-9t | 0 | 0, 14 | 0 | 0, 26 | 0 | 0, 12 | *.039* |
| Oleic acid, 18:1n-9c | 2102 | 1602, 2480 | 4004 | 3137, 5010 | 2183 | 1300, 2951 | *< .001* |
| Cis-vaccenic acid, 18:1n-7 | 99 | 76, 132 | 198 | 166, 268 | 111 | 64, 148 | *< .001* |
| Eicosenoic acid, 20:1n-9 | 16 | 11, 21 | 29 | 20, 36 | 12 | 6, 20 | *< .001* |
| Unknown FA1 | 29 | 22, 36 | 66 | 53, 84 | 40 | 25, 53 | *< .001* |
| Unknown FA2 | 5 | 4, 7 | 10 | 3, 13 | 4 | 2, 7 | *< .001* |
| **MUFA** | **2496** | 1888, 2885 | **4953** | 3972, 6249 | **2729** | 1716, 3542 | ***< .001*** |
| Linoleic acid, 18:2n-6 | 422 | 332, 498 | 807 | 657, 972 | 434 | 243, 540 | *< .001* |
| Linolenic acid (ALA), 18:3n-3 | 22 | 17, 30 | 45 | 31, 59 | 22 | 12, 31 | *< .001* |
| Stearidonic acid, 18:4n-3 | 0 | 0, 3 | 0 | 0, 7 | 0 | 0, 5 | *< .001* |
| Eicosadienoic acid, 20:2n-6 | 0 | 0, 5 | 0 | 0, 6 | 0 | 0, 3 | *.086* |
| Eicosatrienoic acid, 20:3n-6 | 4 | 0, 7 | 9 | 0, 14 | 4 | 0, 9 | *< .001* |
| Arachidonic acid, 20:4n-6 | 8 | 5, 13 | 17 | 12, 25 | 8 | 4, 13 | *< .001* |
| Docosapentaenoic acid (DPA), 22:5n-3 | 0 | 0, 4 | 0 | 0, 7 | 0 | 0, 4 | *< .001* |
| Docosahexaenoic acid (DHA), 22:6n-3 | 0 | 0, 2 | 0 | 0, 3 | 0 | 0, 0 | *.096* |
| **PUFA** | **466** | 373, 552 | **902** | 723, 1096 | **472** | 276, 594 | ***< .001*** |
