# Supplementary material for: Impact of the FTO rs9939609 risk allele on subcutaneous adipose tissue fatty acid composition in adults with obesity class 2 and 3
Source: PLoS One. 2026 Jun 17;21(6):e0351698. doi: 10.1371/journal.pone.0351698 (PMC13274855; doi:10.1371/journal.pone.0351698)
Supplement: S5 Table — (DOCX) [file pone.0351698.s006.docx]

**S5 Table**. **Fatty acid mass content (g per depot) of android and gynoid adipose tissue in females and males**

|  | **Females** | | **Males** | | Android difference | **Females** | | **Males** | | Gynoid difference |
| --- | --- | --- | --- | --- | --- | --- | --- | --- | --- | --- |
|  | Android, n=65 | | Android, n=27 | |  | Gynoid, n=66 | | Gynoid, n=26 | |  |
|  | Median g | 25^th^, 75^th^ percentiles | Median g | 25^th^, 75^th^ percentiles | P-value | Median g | 25^th^, 75^th^ percentiles | Median g | 25^th^, 75^th^ percentiles | P-value |
| Lauric acid, 12:0 | 9 | 0,15 | 10 | 0, 23 | *.412* | 0 | 0, 31 | 0 | 0, 29 | *.908* |
| Myristic acid, 14:0 | 103 | 76, 130 | 132 | 86, 168 | *.031* | 182 | 146, 235 | 148 | 104, 220 | *.016* |
| Pentadecanoic acid, 15:0 | 10 | 7, 12 | 12 | 9, 15 | *.039* | 19 | 16, 26 | 14 | 9, 22 | *.006* |
| Palmitic acid, 16:0 | 892 | 706, 1041 | 1265 | 829, 1391 | *.003* | 1707 | 1389, 1982 | 1345 | 1082, 1696 | *.009* |
| Heptadecanoic acid, 17:0 | 6 | 4, 8 | 7 | 5, 9 | *.038* | 9 | 7, 12 | 8 | 4, 11 | *.270* |
| Stearic acid, 18:0 | 121 | 90, 146 | 147 | 93, 173 | *.040* | 161 | 131, 207 | 134 | 112, 171 | *.010* |
| **SFA** | **1158** | 898, 1352 | **1562** | 1110, 1845 | ***.005*** | **2101** | 1701, 2476 | **1669** | 1347, 2223 | ***. 019*** |
| Myristoleic acid, 14:1n-5 | 11 | 8, 16 | 13 | 8, 22 | *.204* | 30 | 22, 42 | 23 | 18, 39 | *.165* |
| Pentadecenoic acid, 15:1 | 0 | 0, 2 | 1 | 0, 3 | *.420* | 3 | 0, 5 | 0 | 0, 5 | *.581* |
| Palmitoleic acid, 16:1n-7 | 202 | 144, 271 | 231 | 153, 336 | *.090* | 595 | 438, 795 | 492 | 344, 632 | *.010* |
| Elaidic acid, 18:1n-9t | 0 | 0, 10 | 0 | 0, 16 | *.152* | 0 | 0, 25 | 7 | 0, 28 | *.366* |
| Oleic acid, 18:1n-9c | 2005 | 1540, 2299 | 2390 | 1850, 2834 | *.023* | 4354 | 3632, 5231 | 2945 | 2553, 4335 | *<.001* |
| Cis-vaccenic acid, 18:1n-7 | 97 | 74, 119 | 124 | 78, 145 | *.106* | 220 | 177, 282 | 171 | 121, 212 | *<.001* |
| Eicosenoic acid, 20:1n-9 | 16 | 12, 22 | 15 | 10, 21 | *.684* | 32 | 23, 41 | 21 | 12, 29 | *<.001* |
| Unknown FA1 | 29 | 21, 34 | 32 | 25, 38 | *.116* | 71 | 59, 90 | 52 | 39, 65 | *<.001* |
| Unknown FA2 | 5 | 3, 7 | 6 | 4, 8 | *.054* | 10 | 7, 14 | 9 | 6, 12 | *.165* |
| **MUFA** | **2436** | 1828, 2779 | **2820** | 2171, 3409 | ***.023*** | **5413** | 4485, 6439 | **3769** | 2982, 5387 | ***<.001*** |
| Linoleic acid, 18:2n-6 | 409 | 320, 482 | 451 | 381, 548 | *.089* | 882 | 734, 1060 | 622 | 472, 787 | *<.001* |
| Linolenic acid (ALA), 18:3n-3 | 22 | 17, 30 | 23 | 16, 31 | *.850* | 50 | 40, 64 | 32 | 22, 43 | *<.001* |
| Stearidonic acid, 18:4n-3 | 0 | 0, 3 | 0 | 0, 0 | *.444* | 0 | 0, 7 | 0 | 0, 5 | *. 921* |
| Eicosadienoic acid, 20:2n-6 | 2 | 0, 5 | 0 | 0, 4 | *.044* | 0 | 0, 7 | 0 | 0, 3 | *.202* |
| Eicosatrienoic acid, 20:3n-6 | 4 | 0, 8 | 0 | 0, 6 | *.022* | 11 | 0, 17 | 0 | 0, 5 | *<.001* |
| Arachidonic acid, 20:4n-6 | 8 | 5, 13 | 8 | 6, 13 | *.983* | 18 | 13, 28 | 13 | 7, 17 | *.003* |
| Docosapentaenoic acid (DPA), 22:5n-3 | 0 | 0, 4 | 0 | 0, 4 | *.318* | 0 | 0, 8 | 0 | 0, 4 | *.160* |
| Docosahexaenoic acid (DHA), 22:6n-3 | 0 | 0, 2 | 0 | 0, 3 | *.905* | 0 | 0, 4 | 0 | 0, 0 | *.504* |
| **PUFA** | **457** | 358, 547 | **474** | 412, 597 | ***.158*** | **972** | 805, 1203 | **694** | 514, 829 | ***<.001*** |
