# Supplementary material for: Impact of the FTO rs9939609 risk allele on subcutaneous adipose tissue fatty acid composition in adults with obesity class 2 and 3
Source: PLoS One. 2026 Jun 17;21(6):e0351698. doi: 10.1371/journal.pone.0351698 (PMC13274855; doi:10.1371/journal.pone.0351698)
Supplement: Attachment — Submitted filename: Response to Reviewers.docx [file pone.0351698.s008.docx]

Dear Dr. Marwan Salih Al-Nimer

Academic Editor
PLOS One

Thank you for the nice invitation to submit a revised version of the manuscript ‘PONE-D-26-01746’ and in this letter to address the points raised during the review process by the academic editor and Reviewers 1 and 2. We are also very grateful that we were granted additional time to complete the revision.

Please find the following files attached (in the online submission form):

- ‘Revised Manuscript with Track Changes’
- ‘Manuscript’
- Separate files as to the ‘Supporting information’:
  - Inclusivity-in-global-research-questionnaire_PONE_D_26_01746
  - S2 Table revised
  - S4 Table new
  - S5 Table renumbered
- Fig 1, revised to a TIF file
- Fig 2, revised to a TIF file
- Cover letter, revised

We have used standard laboratory protocols for extraction of subcutaneous adipose tissue samples, and gas chromatography (GC-MS) of the Scan Mode to determine fatty acids, so we do not consider it necessary or appropriate to deposit them in protocols.io. It is not applicable (N/A) to publish peer-reviewed Lab Protocol articles.

**Our rebuttal on each request is given by this formatting:**

- text in *italic* = *the original request* from the academic editor, and from Reviewers 1 and 2
- text in quotation marks = “our new text added or changed in the revised manuscript”, supported by references to the line numbers in the ‘Manuscript’ (the ‘clean’ version)

Please get in touch if anything is unclear or incomplete, or needs further explanation, or if we can be of assistance in any other way.

On behalf of the co-authors,

Kind regards

Dr. Ingrid L. Mostad, corresponding author

***Journal requirements:***

*When submitting your revision, we need you to address these additional requirements.*

*1. Please ensure that your manuscript meets PLOS ONE's style requirements, including those for file naming.*

Answer: It’s done.

*2. Please include a complete copy of PLOS’ questionnaire on inclusivity in global research in your revised manuscript.*

Answer: It’s uploaded separately and added to the list of ‘Supporting information’ in the ‘Manuscript’:

“Inclusivity-in-global-research-questionnaire_PONE_D_26_01746”

Please note, that as requested in the questionnaire we have provided more details as to who granted permissions for the study and have given the Norwegian name for the regional ethics committee, see the added and partly revised sentences on lines 72-77:

*“*The study was conducted according to the guidelines laid down in the Declaration of Helsinki and all procedures involving research study participants were approved by the Regional committee for medical and health research in Central Norway, Regional komité for medisinsk og helsefaglig forskningsetikk, Midt-Norge, REK midt, registration number 2013/642. The Clinic of Surgery, St. Olavs hospital – Trondheim University Hospital approved the study. All volunteers provided written, signed informed consent to participate”.

3. *Thank you for stating the following financial disclosure:*

*“This work was supported by the Liaison Committee between the Central Norway Regional Health Authority (RHA) and the Norwegian University of Science and Technology (NTNU); and the Joint Research Committee between St. Olavs hospital and the Faculty of Medicine and Health Sciences, NTNU (FFU). The supporting sources were not involved or had no restrictions regarding publication.”*

*Please state what role the funders took in the study.  If the funders had no role, please state: "The funders had no role in study design, data collection and analysis, decision to publish, or preparation of the manuscript."*

*If this statement is not correct you must amend it as needed.*

Answer: The statement is correct and we have replaced the last sentence with this one: “The funders had no role in study design, data collection and analysis, decision to publish, or preparation of the manuscript”.

*Please include this amended Role of Funder statement in your cover letter; we will change the online submission form on your behalf.*

Answer: Thank you, it is included in our revised cover letter.

*4. Thank you for stating the following in the Funding Section of your manuscript:*

*“: This work was supported by the Liaison Committee between the Central Norway Regional Health Authority (RHA) and the Norwegian University of Science and Technology (NTNU); and the Joint Research Committee between St. Olavs hospital and the Faculty of Medicine and Health Sciences, NTNU (FFU). The supporting sources were not involved or had no restrictions regarding publication.”*

*We note that you have provided funding information that is currently declared in your Funding Statement. However, funding information should not appear in the Acknowledgments section or other areas of your manuscript. We will only publish funding information present in the Funding Statement section of the online submission form.*

Answer: We have deleted the funding information in the ‘Revised Manuscript with Track Changes’, that in the original submitted manuscript was on page 1.

*Please remove any funding-related text from the manuscript and let us know how you would like to update your Funding Statement.*

Answer: We did not have any funding-related text elsewhere in the original submitted manuscript, except for at page 1, as mentioned above.

*5. We note that you have indicated that there are restrictions to data sharing for this study. For studies involving human research participant data or other sensitive data, we encourage authors to share de-identified or anonymized data. However, when data cannot be publicly shared for ethical reasons, we allow authors to make their data sets available upon request. For information on unacceptable data access restrictions, please see http://journals.plos.org/plosone/s/data-availability#loc-unacceptable-data-access-restrictions.*

*Before we proceed with your manuscript, please address the following prompts:*

*a) If there are ethical or legal restrictions on sharing a de-identified data set, please explain them in detail (e.g., data contain potentially identifying or sensitive patient information, data are owned by a third-party organization, etc.) and who has imposed them (e.g., a Research Ethics Committee or Institutional Review Board, etc.). Please also provide contact information for a data access committee, ethics committee, or other institutional body to which data requests may be sent.*

Answer: Some restrictions will apply. The applicable point is that data contain potentially identifying or sensitive patient information and cannot be shared publicly because of Norwegian law that does not open for this type of data sharing. The Regional ethics committee in Central Norway that approved the study- REC Central (REK midt)- will confirm this in a written statement if required.

REC Central can be contacted via e-mail: [rek-midt@mh.ntnu.no](mailto:rek-midt@mh.ntnu.no) , or phone number +47 73597511.

We want to include the statement “available upon reasonable request”.

*b) If there are no restrictions, please upload the minimal anonymized data set necessary to replicate your study findings to a stable, public repository and provide us with the relevant URLs, DOIs, or accession numbers. Please see http://www.bmj.com/content/340/bmj.c181.long for guidelines on how to de-identify and prepare clinical data for publication. For a list of recommended repositories, please see https://journals.plos.org/plosone/s/recommended-repositories. You also have the option of uploading the data as Supporting Information files, but we would recommend depositing data directly to a data repository if possible.*

Answer: N/A

*Please update your Data Availability statement in the submission form accordingly.*

Answer: Data Availability statement in the submission form will be “available upon reasonable request”.

*6. We note that you have included the phrase “data not shown” in your manuscript. Unfortunately, this does not meet our data sharing requirements. PLOS does not permit references to inaccessible data. We require that authors provide all relevant data within the paper, Supporting Information files, or in an acceptable, public repository. Please add a citation to support this phrase or upload the data that corresponds with these findings to a stable repository (such as Figshare or Dryad) and provide and URLs, DOIs, or accession numbers that may be used to access these data. Or, if the data are not a core part of the research being presented in your study, we ask that you remove the phrase that refers to these data.*

Answer: The first wording “(data not shown)” is replaced with (“**Table 4”**), on line 218. This Table 4 is a new table, corresponding to the similar Table 2. The former Table 4 is renumbered to be Table 5. The new Table 4 is set in the ‘Manuscript’ just before the renumbered Table 5.

The second wording “(data not shown)” is replaced with (“**S4 Table”**), on line 245. This S4 Table is a new table, corresponding to the similar S2 Table. The former S4 Table is renumbered to be the S5 Table. The new S4 Table is submitted separately as Supporting Information, as is the revised S5 Table. In addition, the title of the new table is added on the list of Supporting information on lines 429-439 in the ‘Manuscript’: “**S4 Table. Fatty acid mass content (g per depot) of android and gynoid adipose tissue, all participants**”.

*If the reviewer comments include a recommendation to cite specific previously published works, please review and evaluate these publications to determine whether they are relevant and should be cited. There is no requirement to cite these works unless the editor has indicated otherwise.*

Answer: N/A

***Reviewers' comments:***

Our comment: The Reviewers’ comments 1.-4. do not require a response from us, so we will start with point 5.

*5. Review Comments to the Author*

***Reviewer #1:*** *The study involves a very large number of statistical comparisons across genotypes,* *sexes, depots, individual fatty acids, and grouped fatty acids, which substantially increases the risk of false-positive findings. Although Bonferroni correction is applied in selected analyses, the overall analytical strategy remains highly exploratory. This concern is particularly relevant for the male subgroup analyses, where sample sizes are very small (e.g., n = 6 in the TT genotype group). While the authors appropriately express caution in the Discussion, this exploratory nature should be emphasized more explicitly in the Results, Abstract, and Conclusions to avoid overinterpretation by readers?*

Answer: Thank you for this justified comment, firstly addressed by adding the following statement on line 27 in the Abstract: “We interpret these findings with caution due to the small numbers of males with the TT genotype”.

Secondly, we have added the following caveat in Results, on lines 157-158: “The group of males with the non-risk TT genotype is very small (n=6), which means that their results must be interpreted with caution”.

Thirdly, in the conclusions we have added on lines 340-341: “…, especially because these results occur in the male genotype group with the fewest number of participants”.

*The numerous tables and highly detailed reporting of each fatty acid may obscure the main findings. The authors should simplify the Results section by focusing on key biological patterns and adding brief summaries to help readers navigate the data. Despite these concerns, the manuscript makes a valuable contribution.*

Answer: Thank you for this valuable and nice comment.

To help focus on the main results, we have revised the subheadings to match the table titles, and inserted two additional subheadings, so that each table is mentioned in text sections of corresponding and logical subheadings. The slightly revised subheadings are on lines 167 and 214, and the new subheadings on lines 191 and 243.

We have added brief summaries on the following lines:

Lines 177-179, a result summary of “Effect of genotype on FA composition (weight %)”

Lines 209-213, a result summary of “Effect of depot on FA composition (weight %)”

Lines 226-228, a result summary of “Effect of genotype on FA mass content (g per depot)”

Lines 254-258, a result summary of “Effect of depot on FA mass content (g per depot)”

***Reviewer #2:*** *The study titled “Impact of the FTO rs9939609 risk allele on subcutaneous adipose tissue fatty acid composition in adults with obesity” by Mostad et al addresses an important and relevant topic; however, several revisions are needed to improve the clarity and rigor of the manuscript.*

*1. Pages 9-10. The introduction would benefit from a clearer and more explicitly defined research gap early in the section. While the authors reference several potential gaps, including non-satiety mechanisms, fatty acid metabolism, and depot-specific effects, the primary unanswered question is not clearly articulated. A more focused statement outlining exactly what remains unknown and how the present study addresses this gap would improve clarity and strengthen the framing of the manuscript.*

Answer: We are thankful for the justified comments from Reviewer 2. Each of the four paragraphs of the introduction has therefore been thoroughly revised, and the changes that can be traced in the ‘Revised Manuscript with Track Changes’ can be summarized as follows, when referring to the line numbers of the clean ‘Manuscript’:

Lines 35-40: Non-satiety mechanisms are elaborated, and two more references are added to this paragraph.

Lines 41-48: Potential effects of *FTO* on lipid and FA metabolism in adipose tissue are elaborated, and some mechanisms and new references are added. In all six references are given in this paragraph, but none of our own research, so far.

Lines 49-58: Genetic effects on metabolism not related to BMI and affected by the *FTO* obesogenic rs9939609 risk allele are presented (including our own studies but without any details), stating that any coupling between the risk allele to the adipose tissue composition of FA has so far not been tested, leading up to the statement that it is relevant to investigate that by taking into account the size of specific depots of adipose tissue.

Lines 59-62: The last paragraph now states a hypothesis and the aim.

*2. Pages 9-10: A substantial portion of the introduction summarizes the authors’ prior studies (refs 6–10). While this work is relevant, the emphasis creates a somewhat self-referential tone and limits broader contextualization within the existing literature. Expanding the discussion to more comprehensively synthesize findings from independent groups would provide a more balanced and field-wide perspective.*

Answer: Thank you for these valuable comments. Instead of summarizing our own prior studies in the Introduction, this is moved to the beginning of the Discussion. Importantly, we have given a broader contextualization within the existing literature and added other references, see summary of the revisions of the first three on lines 35-58 in the ‘Manuscript’.

*3. Pages 9-10: The biological rationale underlying the study requires further development. Specifically, the proposed link between FTO, fatty acid metabolism, and adipose tissue fatty acid composition is introduced but not convincingly explained. Additionally, the rationale for examining fatty acid composition rather than adiposity measures themselves should be more clearly justified.*

Answer: Thank you for pointing out these important matters, which we have sorted out in the revised introduction on lines 35-58 in the ‘Manuscript’.

*4. Line 69: The inclusion criteria specify participants aged ≥20 years, but no upper age limit is reported. Please clarify whether an upper age cutoff was applied, as advanced age can substantially influence adipose tissue biology and fatty acid composition and may confound the interpretation of obesity-related metabolic findings.*

Answer: Thank you for this valuable comment. There was no upper age limit for inclusion in the study, providing the participants were able to carry out the study. The oldest participant was 66 y old, and the median age was 42 y.

We have deleted the former statements on lines 289-290 in the originally submitted version (“We see the adult participants’ comparatively young age as an advantage. This is because the *FTO* locus gradually loses its importance for the development of obesity with increasing age (21, 22)”).

The age discussion is moved to the limitations section and elaborated, as follows on lines 329-334:

“We acknowledge that advanced age can influence tissue biology and FA composition (27) and may confound the interpretation of obesity-related metabolic findings (28, 29). However, these studies did not investigate the *FTO* locus related to the FA composition of adipose tissue. Their conclusion was that the *FTO* effect on body composition appeared to be weakened with age, which could also have had an impact on our results, even though our participants had a median age of 42 y”.

*5. Line 69: The study includes participants with BMI ≥35 kg/m², whereas standard definitions classify obesity at BMI ≥30 kg/m². Please clarify the rationale for using this higher threshold.*

Answer: Thank you for the request. The obesity clinic accepted referrals for adults with BMI 35-39.9 kg/m² (obesity class 2) if they had weight-related comorbidities. Without known comorbidities, BMI must be ≥40 kg/m² (obesity class 3). This is in line with the Norwegian Health Directory guidelines (2011). Because we recruited from this clinic, our participants had BMI ≥35 kg/m².

For clarity, we have added “class 2 and 3” in the paper title on line 2: ‘Impact of the *FTO* rs9939609 risk allele on subcutaneous adipose tissue fatty acid composition in adults with obesity “class 2 and 3”. By the same reason we have added as follows in Material and methods on lines 67-68: ‘Individuals that were recruited between 21st August 2013 and 1st September 2015 had newly been referred to St. Olavs hospital outpatient obesity clinic “for adults with obesity class 2 or 3”.

*6. Page 33: The analysis focuses on a single FTO locus (rs9939609). However, other FTO variants may have distinct or even divergent effects on adiposity and body composition. Please acknowledge this limitation and discuss how restricting the analysis to one locus may influence the interpretation of the genetic findings.*

Answer: Thank you for your request. In our opinion it is an assumption that other *FTO* variants may have distinct or even divergent effects on adiposity and body composition. Therefore, we have added the following sentences in the section of limitations on lines 334-336: “The use of a single risk allele probe, here rs9939609, constitutes a theoretical limitation; however, rs9939609 has been employed in multiple other studies and, after searching the literature, we find no evidence that results with rs9939609 are contradictory to results using other probes”.

*7. Page 33: The study does not evaluate mRNA expression levels across FTO rs9939609 genotypes or assess appetite- and hormone-related markers. Given that variation in FTO expression and associated hormonal pathways may influence body composition, the absence of these functional correlates should be acknowledged.*

Answer: Thank you for the valuable comments. We have added the following in the limitations section in the Discussion on lines 328-329: “Another limitation is the lack of gene expression data of the *FTO* locus in the current study”.

Regarding appetite-related hormones, they were evaluated in a separate publication in PLOS One in 2025, now referred to in the Discussion on lines 262-264: “On the other hand we found no clear association of the risk allele with self-reported dietary intake including SFA, MUFA and PUFA (17), or with appetite-related hormones (18)”.

Additional information to the editor.

As announced in the Cover letter, we have made some minor revisions for the sake of clarity, as listed:

1. Lines 152, 194, 197 and 245: In the brackets “(**S1 Table**)”, “(**S2 Table**)”, “(**S3 Table**)” and “(**S4 Table**)” the tables’ titles were written out in full in the originally submitted paper (on lines 148, 185-186, 189-190 and 229-230). We deleted the titles in the brackets because we considered them to be unnecessary, since they were listed in the ‘Supporting information’.

2. On line 284 we deleted a reference that was incorrectly inserted after “(S2 Table)”.
3. We had forgotten to state that all biological material in our study is registered in a public biobank. Therefore, we added the following information on lines 101-102: “Biological samples in our study were registered in the official biobank in Central Norway, Biobank1®”.
4. We realized that the information given in Table 1, footnote a), implicated that the number of participants who had results varied by one person from FA results on weight % compared with g per depot, with no specific information about that in the submitted paper. Therefore, we added the following information on lines 126-129: “Because we lacked the DXA scan of fat mass variables from one participant, the calculation of each FA as g per depot is given for one participant less, than for the FA expressed as weight %. The correct number of participants is clearly marked in the header rows for each relevant table”.
5. We discovered that while the participant numbers were correct in most, they were not in all affected tables, so some corrections were needed. Therefore, the header rows in Table 2 of the manuscript were revised, as shown in ‘Revised Manuscript with Track Changes’. The header rows for S2 Table were also revised, but without tracking the changes, as they are submitted as ‘Supporting information’.
